# A Trade-off between Force and Flow May Lead to Reduced Entropy Production Rate during Faster Microbial Growth

**DOI:** 10.1021/acs.jpcb.4c08559

**Published:** 2025-06-06

**Authors:** Maarten J. Droste, Maaike Remeijer, Robert Planqué, Frank J. Bruggeman

**Affiliations:** † Department of Mathematics, Amsterdam Center for Dynamics and Computation, 1190 Vrije Universiteit Amsterdam, Amsterdam, 1081 HV, The Netherlands; ‡ Systems Biology Lab, A-LIFE, AIMMS, Vrije Universiteit Amsterdam, Amsterdam, 1081 HZ The Netherlands

## Abstract

Thermodynamics dictates that the entropy production rate (EPR) of a steady-state isothermal chemical reaction network rises with reaction rates. Living cells can, in addition, alter reaction rates by changing enzyme concentrations, giving them control over metabolic activities. Here, we ask whether microbial cells can break this relation between EPR and reaction rates by shifting to a metabolism with lower thermodynamic driving force (per unit of biomass) at faster growth. First, we study an example metabolic network to illustrate that maximization of metabolic flux by optimal allocation of resources can indeed lead to selection of a pathway with a lower driving force. This pathway then compensates for the reduction in driving force by relying on fewer enzymes with sufficiently increased concentrations, resulting in a higher flux. Next, we investigate the EPR per unit biomass of microbes that change their catabolic network as a function of their growth rate, using three models for chemostat cultivation of the yeast *Saccharomyces cerevisiae* that are calibrated with experimental data. Although current experimental evidence proved insufficient to give conclusive results, we derive a general criterion to predict when the specific EPR drops after a metabolic switch. We describe the experiments that are required to show that the specific EPR of a microbe can decrease with its growth rate.

## Graphical abstract

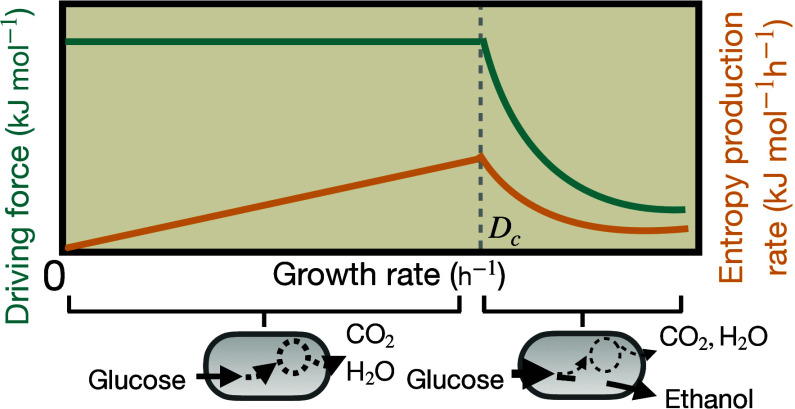

## 1 Introduction

Living systems such as growing (microbial) cells, multicellular systems and ecosystems are ‘open’ thermodynamic systems.^1^ These systems maintain displaced from thermodynamic equilibrium by a continuous exchange of mass and energy with their environment. They can attain nonequilibrium steady-states (NESSs) at constant conditions, which are characterized by a net entropy production. The production of entropy by open systems prevents them from reaching their maximal entropy state—the thermodynamic equilibrium state—and continues as long as their driving force has not been depleted. Out-of-equilibrium, open systems therefore effectively feed on ‘negentropy’,^2,3^ when they produce entropy.

Thermodynamics plays an important role in microbial physiology. For instance, modes of microbial growth are, for instance, distinguished by the free-energy source for synthesis of energy equivalents (ATP, NAD­(P)­H) and precursors (e.g., amino acids, nucleic acids) for biosynthesis of macromolecular constituents (proteins, D/RNA, etc.).^4^ Novel modes of catabolism have even been discovered from the existence of net driving forces in nature^5^ (e.g., anaerobic ammonium oxidation^6^), as captured by the famous statement of Baas Becking: “The environment selects” (with environment interpreted as the ‘driving force’).^7^ Thermodynamics has been used to analyze the efficiency of energy transduction from catabolism to anabolism^8^ and offers, in principle, a method to do so without having to consider reaction kinetics, which are generally poorly known. The “energy-converter” model has in particular received ample attention.^8,9^ Most of its insights are, however, only applicable close to thermodynamic equilibrium, when reaction rates depend linearly on their driving force, and the entropy production is minimal.^1^ Although this does occur in microbial physiology,^10^ it is not the general case. More recently, (classical) thermodynamic approaches have been integrated with genome-scale modeling to estimate formation energies and ATP requirements for biomass synthesis,^11^ and to predict the entropy production of microbial growth on different carbon sources.^12^

Balanced growth,^13^ a state in which a culture of cells grows at a fixed rate and cellular metabolism is in steady state, is an example of a NESS^14,15^ and has been of major importance in the development of microbial physiology as a discipline. Balanced growth can be attained with continuous culturing techniques,^16^ such as the chemostat,^17^ which allow for the prolonged cultivation of microbes under NESS conditions.

The entropy production rate (EPR) of metabolism operating under NESS conditions (i.e., at balanced growth) has been investigated. Theoretical studies exist that express the EPR of a metabolic network as a sum of the products of the steady-state rates and driving forces of its reactions.^15,18,19^ Some have speculated that evolution of microbial growth proceeds in the direction of an increased EPR^20^ while others have argued that rates of microbial metabolism and growth are bounded by a maximal EPR.^21^ Saadat et al.^11^ has examined the potential relationship between thermodynamic limits and the optimality of microbial growth. Similar discussions exists for physical and chemical systems,^22,23^ evolution of biological complexity^24^ and functioning ecosystems.^25^ However, since no fundamental proofs exists about the EPR of NESSs (as a function of rate) the general validity of the statements remain to be shown. Counterarguments should therefore come from concrete biological examples, showing that a certain case can indeed occur – even though this can not (yet) be proven from fundamental principles.

In this paper, we carry out a novel investigation of a thermodynamic aspect of microbial physiology. We aim to study the EPR per unit of biomass, i.e., the specific entropy production rate (sEPR), of a culture of microbial cells, which is maintained in a NESS in a chemostat, as a function of its growth rate. This is motivated by a recent interest in systems biology on understanding why many microbes shift from a mode of metabolism with a high biomass yield to another low-yield mode as a function of their growth rate in a chemostat. Here we ask how this shift influences the sEPR of microbial growth. In particular, can the sEPR decrease as a function of the growth rate when the microbe changes to a metabolism with lower thermodynamic driving force (per unit of biomass)?

For instance, at high growth rates in an aerobic glucose-limited chemostat, *S. cerevisiae* and *E. coli* gradually shift metabolism from pure respiration toward overflow metabolism.^26–29^ Overflow metabolism involves the partial degradation of glucose into ethanol or acetate and yields less ATP per glucose than respiration. It is likely that the partial degradation of glucose occurs at a lower driving force than respiration. This hypothesis is supported by other findings^30^ for the catabolic part of the metabolic network. Even though overflow metabolism occurs at a higher rate, its sEPR can then still be lower than during respiration occurring at a lower growth rate. This would go against intuition, since reaction rates increase with the driving force, the sEPR which equals the product of the rate of driving force for all cellular reactions is expected to increase with cellular growth rate as well, as this is accompanied by an increase of all reaction rates. This intuition is in particular due to our understanding of the behavior of entropy production rate of chemical reaction networks.^31,32^ Since biochemical reaction networks depend on enzyme-catalyzed reactions, and living cells can change those enzyme concentrations as a function of conditions and alter the length of their metabolic pathways, e.g. during metabolic shifts, it remains unclear how the sEPR of microbial cells depends on their growth rate.

We show that the chemostat is a suitable device to study these problems, as the net driving force and rate of growth can be independently determined, allowing for a calculation of the sEPR. Using a combination of stoichiometric and chemostat models, we explore the possibility that sEPR does not increase with growth rate after a metabolic switch, and derive a sufficient condition for the sEPR to decrease after such a switch. These results indicate that despite feeding on negentropy to stay away from thermodynamic equilibrium, higher growth rates do not necessarily involve higher entropy production rates.

## 2 Theory

### 2.1 Reaction Kinetics and Stoichiometric Network Theory

In this section, we first briefly summarize two pieces of existing theory before we integrate them. We relate theory about (bio)­chemical reactions, their stoichiometry, kinetics and thermodynamics^15,18,33^ to theory about steady-state flux distributions of reaction networks and how they decompose into elementary flux vectors.^34^ The combined theory describes the EPR of a reaction network in terms of its stoichiometric structure and kinetics. This will enable us to contrast chemical reaction networks that lack reaction-catalyzing enzymes with biochemical networks that do have them.

### 2.2 Chemical versus Biochemical Reaction Networks

Chemical reactions differ from enzyme-catalyzed biochemical reactions. Chemical reactions occur spontaneously without catalytic agents; the rate of a biochemical reaction, at constant temperature and pressure, depends on the concentration of the reactants, setting the reaction’s thermodynamic driving force, and the concentration of the catalyzing enzymes and (allosteric) effectors.^35^ (The enzyme is a catalyst because it does not change during the conversion; it is unaltered by the catalytic events.) In contrast, a chemical reaction depends only on the driving force, which is set by its reactant concentrations.

The stoichiometry of any (bio)­chemical reaction occurring in a network can be written as

$$\overset{n_{C}}{\underset{i=1}{\sum }}n_{ij}^{+}C_{i}\overset{\mathrm{reaction}j}{⇌}\overset{n_{C}}{\underset{i=1}{\sum }}n_{ij}^{-}C_{i}$$

with *C*_*i*_ as the name of chemical compound *i*, *n*_*C*_ as the number of reactants in the network, *n*_*ij*_^+^ (≥0) as the substrate stoichiometric constant of *C*_*i*_ in reaction *j*, *n*_*ij*_^–^ (≥0) as the product stoichiometric constant of *C*_*i*_ in reaction *j*. The net stoichiometric coefficient of reactant *i* in reaction *j* equals $n_{ij}=n_{ij}^{-}-n_{ij}^{+}$ ; this coefficient enters in the stoichiometric matrix. The net stoichiometric coefficient for an enzyme is zero, for a substrate it is negative and for a product it is positive.

In well-mixed conditions, the rate *v*_*j*_ of chemical reaction *j* obeys mass-action kinetics

vj(c′)=vj+(c′)−vj−(c′)=kj+∏i=1nCcinij+−kj−∏i=1nCcinij−=kj+∏i=1nCcinij+(1−∏i=1nCcinij−Keq,j∏i=1nCcinij+)=vj+(1−vj−/vj+) 1

The concentration of *C*_*i*_ is denoted here as *c*_*i*_, *k*_*j*_^+^, and *k*_*j*_^–^ are, respectively, the forward and the backward rate constant, and *K*_eq,*j*_ is the equilibrium constant of reaction *j*. Here, we assumed that the rate constants are independent of the state of the system. Hereby, we exclude any effects of the reactants’ concentrations on the medium properties. The vector **c**′ is the vector **c**, containing the concentrations of the time-dependent reactants, augmented with the concentrations of the reactants that are held fixed (they are thus seen as ‘external’ to the (bio)­chemical reaction network).

The rate *v*_*j*_ of an enzyme-catalyzed biochemical reaction *j* (that also obeys mass-action kinetics) can generally be written as,^14^

2 vj(c′)=vj+(c′)−vj−(c′)=kcat,j+ejfj+(c′)−kcat,j−ejfj−(c′)=kcat,j+ejfj+(c′)(1−∏i=1nCcinij−Keq,j∏i=1nCcinij+)=vj+(1−vj−/vj+)

with $k_{\mathrm{cat},j}^{+}$ and $k_{\mathrm{cat},j}^{-}$ as the forward and backward catalytic rate constants, $0\leq f_{j}^{+}\left(c^{\prime }\right)\leq 1$ $\left(0\leq f_{j}^{-}\left(c^{\prime }\right)\leq 1\right)$ as the substrate (product) saturation function (which can contain activating or inhibiting (allosteric) effector influences, enzyme cooperativity, etc.) of enzyme *j* catalyzing reaction *j*. Note that the rate of an enzymatic reaction depends on the concentration of reactants and effectors (in **c**′) and on the enzyme concentration. Effector and enzyme concentrations do not play a role in chemical reaction networks.

The term that occurs in both chemical and biochemical reaction kinetics,

3 $$1-\frac{\overset{n_{C}}{\underset{i=1}{\prod }}c_{i}^{n_{ij}^{-}}}{K_{\mathrm{eq},j}\overset{n_{C}}{\underset{i=1}{\prod }}c_{i}^{n_{ij}^{+}}}$$

is the displacement from thermodynamic equilibrium by reaction *j*. Below we show that this term relates to the thermodynamic driving force of the reaction, as previously highlighted by Noor et al.^33^

The stoichiometric matrix of a chemical reaction network **N** with entries {**N**}_*ij*_ = *n*_*ij*_ contains all the net stoichiometric coefficients of the reactants with variable (i.e., time-dependent) concentrations.

The rate of change in the concentration of the reactants is now given by

ċ=Nv

and steady state implies

Nv=0

### 2.3 Reaction Thermodynamics

The (molar) Gibbs free energy potential of reaction *j* equals,

$$\Delta \mu_{j}=\overset{n_{C}}{\underset{i=1}{\sum }}n_{ij}\mu_{C_{i}}$$

where $\mu_{C_{i}}$ is the Gibbs free energy per mole of compound *C*_*i*_,^15^

$$\mu_{C_{i}}=\mu_{C_{i}}^{{{}^{\circ}}^{\prime }}+RT\ln c_{i}$$

$\mu_{C_{i}}^{{{}^{\circ}}^{\prime }}$ is the Gibbs free energy of formation per mole under standard conditions,[Fn fn1] *R* is the ideal gas constant and *T* is the temperature.

When reaction *j* operates at thermodynamic equilibrium, Δμ_*j*_ = 0, such that

$$K_{eq,j}=e^{-\Delta \mu_{j}^{o^{\prime }}/RT}$$

where $\Delta \mu_{j}^{{{}^{\circ}}^{\prime }}=\overset{n_{C}}{\underset{i=1}{\sum }}n_{ij}\mu_{C_{i}}^{{{}^{\circ}}^{\prime }}$ is the standard (molar) Gibbs energy of a reaction under standard conditions.[Fn fn2] This leads to a relation for the rate of a (bio)­chemical reaction *j* in terms of its Gibbs free energy potential Δμ_*j*_, via the displacement from thermodynamic equilibrium term ([Disp-formula eq3]),^33^

4 $$1-\frac{\overset{n_{C}}{\underset{i=1}{\prod }}c_{i}^{n_{ij}^{-}}}{K_{\mathrm{eq},j}\overset{n_{C}}{\underset{i=1}{\prod }}c_{i}^{n_{ij}^{+}}}=1-e^{\Delta \mu_{j}/RT}=1-\frac{v_{j}^{-}}{v_{j}^{+}}$$

Thus, at thermodynamic equilbrium: $\frac{\overset{n_{C}}{\underset{i=1}{\prod }}c_{i}^{n_{ij}^{-}}}{K_{\mathrm{eq},j}\overset{n_{C}}{\underset{i=1}{\prod }}c_{i}^{n_{ij}^{+}}}=1$ , Δμ_*j*_ = 0, $v_{j}^{+}=v_{j}^{-}$ and *v*_*j*_ = 0. Note that sign­(*v*_*j*_) = – sign­(Δμ_*j*_). Relation ([Disp-formula eq4]) holds for general kinetics, as investigated in depth by Beard and Qian.^36^

### 2.4 The Rate of a (Bio)­Chemical Reaction Rises with Its Driving Force

Mass-action kinetics ([Disp-formula eq1]) and enzyme kinetics ([Disp-formula eq2]) both depend on the Gibbs free energy potential of the reaction via the term shown in eq 3. Since the (molar) Gibbs free energy potential of a reaction is determined by the concentration of the reactants, a change in the concentration of reactants changes the free energy potential as well as the substrate saturation function in enzyme kinetics. We define *X*_*j*_ = – Δμ_*j*_ as the thermodynamic driving force of the reaction (classical literature defines this differently, i.e. as −Δμ_*j*_/*T* with *T* as temperature, while – Δμ_*j*_ is typically referred to as the chemical affinity^18^). As sign­(*v*_*j*_) = sign­(*X*_*j*_), this is a more convenient quantity to consider in this paper. Since the substrate saturation function of an enzyme generally rises with higher substrate and lower product concentrations, and because this also increases the driving force of the reaction, the substrate saturation function generally rises with the driving force too. Hence, the rate of a (bio)­chemical reaction generally rises with its driving force. (An exception to this relation could be reactions with substrate inhibition. In such cases, the reaction rate may decrease with increasing driving force.)

### 2.5 The Net Conversion and Entropy Production Rate of a Steady-State Reaction Network

We consider a reaction network at a steady state, a NESS. Steady states are achieved by keeping some reactant concentrations fixed at values that lead to a net, nonzero thermodynamic driving force of the network. Each steady-state reaction network brings about a net conversion of ‘substrate’ into ‘product’ reactants. The concentrations of these compounds then eventually determine the Gibbs free energy potential of the entire chemical reaction network, as dictated by Hess’s law.^18^ The direction of the net conversion is such that the total free energy of the products is lower than that of the substrates, i.e. its driving force is positive.

Any steady-state flux vector **v** satisfies **Nv** = **0**. As illustrated in eqs 1 and 2, reversible reactions may be split into two irreversible ones, and the stoichiometric matrix may be extended so that each column corresponds with either a forward or a backward reaction.^34^ We may thus assume by suitably extending **N** that all flux vectors have nonnegative entries. The set {**v**|**Nv** = **0**, *v*_*j*_ ≥ 0} forms a pointed polyhedral cone and is spanned by its extreme rays:^37^ any flux vector can be written as a linear combination of extreme rays using only nonnegative coefficients. Such linear combinations are called conic combinations. In the context of metabolic pathways, the extreme rays are called Elementary Flux Modes (EFMs).^38^ The EFMs are uniquely determined by **N** alone.

A key property of EFMs is that each reaction in the EFM is essential to carry a steady-state flux through the entire EFM. This means that EFMs cannot be further decomposed into simpler networks that sustain a steady-state flux, and that the ratios of flux values in an EFM are constant (and determined by stoichiometry). They are ‘one-degree-of-freedom’ vectors, in the sense that one flux value determines them all. This allows for normalization of an EFM with respect to one of its entries, a property that is useful for this work.

Denoting the steady-state flux vector of a network by **v**(**c**′), we thus have

5 $$v=\overset{N_{\mathrm{EFMs}}}{\underset{i=1}{\sum }}a_{i}E_{i}$$

with *a*_*i*_ ≥ 0 and **E**_*i*_ as the flux vector of EFM *i* (hence, **NE**_**i**_ = 0 as well).

We introduce the stoichiometric matrix **N**′, which equals **N** augmented with the stoichiometric coefficients of the reactants that are held fixed in concentration. For each such a fixed reactant, a new row is added to **N**. The net conversion of the reaction network is referred to as the macrochemical equation (ME).^39^ It is used in microbial physiology, specifically in thermodynamic and yield analyses. It can be obtained from

6 $$\mathrm{ME}=C^{\prime T}N^{\prime }j$$

where **j** is obtained by dividing **v** by one of its entries; it then contains the number of moles of reactants consumed and/or produced (i.e., yields), per mole of one the other reactions. **C**′ is the vector with the names of the reactants, including the fixed ones. The macrochemical equation can for instance equal ME = 5*R* + *S* – 3*P* – 2*Q*, which corresponds to the net reaction 3*P* + 2*Q* → 5*R* + *S* in which 3 mol of *P* and 2 mol of *Q* are converted (directly or through a reaction network) into 5 mol of *R*, per mole of *S*.

The Gibbs free energy potential of the steady-state chemical reaction network can also be obtained from the Gibbs free energies of the reactants,^19^

7 $$\Delta \mu_{\mathrm{net}}=\mu_{C^{\prime }}{\left(c^{\prime }\right)}^{T}N^{\prime }j$$

with μ_**C′**_(**c**′) as the vector of the Gibbs free energies per mole of all the compounds. This is Hess’s law formulated in terms of Gibbs energies.^18^ For the example ME just discussed, the Gibbs free energy potential of the (‘net’) chemical reaction network would thus be Δμ_net_ = 5 μ_R_ + μ_S_ – 3 μ_P_ – 2 μ_Q_. Its driving force equals *X*_net_ = – Δμ_net_.

The vector **j** is closely related to the flux vector **v**. For an EFM, we may in fact substitute **j** by **v**, normalized appropriately. For example, the reaction scheme

A→2B,⁣B→2C,⁣C→D

has a net reaction *A* → 4*D* and EFM equal to (1 2 4)^*T*^, which is uniquely determined up to a constant. Thus, we take **j** = (1 2 4)^*T*^, so that *A* → 4*D* corresponds to Δμ_*net*_ = 4 μ_*D*_ – μ_*A*_.

When EFMs are conically combined as in ([Disp-formula eq5]) the situation is more involved. The exact conic combination depends not only on the concentration of external substrates and products, but also on the internal reaction rates. Consider the simple branched scheme

A→2B,⁣B→C,⁣B→2D

in which *B* is converted into *C* or into *D* (or both). There are two EFMs, **E**_1_ = (1 2 0)^*T*^ and **E**_2_ = (1 0 2)^*T*^, and two corresponding macrochemical equations,

$${\mathrm{ME}}_{1}=-A+2C,,{\mathrm{ME}}_{2}=-A+4D$$

The steady state flux is a conic combination

$$v=a_{1}E_{1}+a_{2}E_{2}$$

where *a*_1_ and *a*_2_ depend at least on the steady state concentrations of *A*, *B*, *C*, and *D* and reaction parameters; if the reactions are additionally catalyzed by enzymes, their concentration also determines the *a*_*i*_’s. We can therefore not deduce a priori the molar quantities of *C* and *D* that are produced per mole of substrate *A* consumed: these quantities depend on the *a*_*i*_. Normalizing on one mole of *A* consumed, we find that the *ME* is

ME=a1a1+a2ME1+a2a1+a2ME2=−A+a1a1+a22C+a2a1+a24D

More generally, introducing the convex coefficients α_*i*_ by normalizing the *a*_*i*_ to sum to 1,

8 $$\mathrm{ME}=\overset{N_{\mathrm{EFMs}}}{\underset{i=1}{\sum }}\alpha_{i}\left(c^{\prime }\right){\mathrm{ME}}_{i},,{\mathrm{ME}}_{i}=C^{\prime T}N^{\prime }j_{i}$$

and

9 $$\Delta \mu_{\mathrm{net}}=\overset{N_{\mathrm{EFMs}}}{\underset{i=1}{\sum }}\alpha_{i}\left(c^{\prime }\right)\Delta \mu_{\mathrm{EFM},i},,\Delta \mu_{\mathrm{EFM},i}=\mu_{C^{\prime }}{\left(c^{\prime }\right)}^{T}N^{\prime }j_{i}$$

The entropy production rate (EPR) Φ of a reaction network is equal to the sum of the products of the driving forces and the rates of its reactions (expressed in moles per unit of time).^15,18^ By applying the EFM decomposition ([Disp-formula eq5]), this can be written for a reaction network consisting of *r* reactions as (with *S* as the entropy)

10 Φ(c′)=dSdt=∑j=1r−Δμj(c′)Tvj(c′)=1TX(c′)Tv(c′)=1T∑i=1NEFMsai(c′)X(c′)TEi(c′)=1T∑i=1NEFMsai(c′)Φi(c′),⁡⁡Φi(c′)=X(c′)TEi(c′)

The steady-state EPR of a chemical reaction network is the weighted sum of the entropy production rates Φ_*i*_(**c**′) of its EFMs. This is the main quantity of interest in this work.

The existing theory summarized here serves as the theoretical basis for our study. In the following section, we place this theory in the context of microbial growth in a chemostat.

### 2.6 Modeling Microbial Growth and the Entropy Production Rate in a Chemostat

A chemostat is a continuously stirred bioreactor operated at a constant volume.^16^ Fresh medium with nutrients flows into the bioreactor at the same rate as medium plus biomass is removed from the bioreactor. Only when the growth rate can compensate for the loss of biomass by outflow can a microbial culture sustain itself in the bioreactor. Because inflow (not containing biomass) and outflow (containing biomass) occur at the same rate *F*, the vessel has a constant volume *V* and a dilution rate *D* = *F*/*V*. The growth rate λ of the microbe needs to equal *D* in order for a steady state (a NESS) to settle.

Microbial growth in the chemostat is generally modeled by condensing the process of cellular growth to the macrochemical equation ([Disp-formula eq6]).^16^ This reaction specifies the overall conversion of nutrients into biomass and (fermentation) products, and its rate, being the growth rate, can be described by a suitable rate equation (e.g., the Monod equation). This is rather straightforward in a chemostat, because this rate is then determined only by a single nutrient concentration called the growth-limiting substrate.

The macrochemical equation can either be obtained directly from experiments, or from a flux vector that is predicted from flux balance analysis of a genome-scale stoichiometric model, as in eq 6. An example that we will be used later, see eq 20, is

$$1.69C_{6}H_{12}O_{6}+0.16{\mathrm{NH}}_{3}+0.012H_{3}{\mathrm{PO}}_{4}+0.0034H_{2}{\mathrm{SO}}_{4}\to {\mathrm{CH}}_{1.61}O_{0.56}N_{0.16}P_{0.012}S_{0.003}+2.20C_{2}H_{6}O+0.73C_{3}H_{8}O_{3}+2.59{\mathrm{CO}}_{2}+0.11H_{2}O$$

The first ‘compound’ on the right-hand side of the reaction is the chemical composition of biomass, expressed per *C*-mole. The macrochemical equation is normalized to the production of 1 *C*-mole of biomass, and thus generally has the form^39^

11 $$\underset{k}{\sum }Y_{S_{k}/B}S_{k}\to B+\underset{l}{\sum }Y_{P_{l}/B}P_{l}$$

The coefficients *Y*_*i*/*B*_ are the yields of compounds *C*_*i*_ per *C*-mole biomass (and hence the entries of the vector **j** discussed above). If growth occurs at a rate λ, the corresponding uptake or excretion rate of compound *i* is now defined as *q*_*i*/*B*_ = *Y*_*i*/*B*_λ.

To model the dynamics of the chemostat we extend its well-known model description^16^ with thermodynamics. The biomass concentration *b* changes due to growth and dilution as

12 ḃ=λb−Db

Nutrients *S*_*k*_ enter the vessel from the reservoir medium at concentrations *s*_*R*,*k*_ and decrease in concentration through outflow and consumption,

13 $$\overset{˙}{s_{k}}=Ds_{R,k}-Ds_{k}-Y_{S_{k}/B}\lambda b$$

Products *P*_*l*_ are excreted by cells at rate $Y_{P_{l}/B}\lambda b$ and flow out,

14 $$\overset{˙}{p_{l}}=Y_{P_{l}/B}\lambda b-Dp_{l}$$

A standard implementation of the growth rate uses the Monod equation,

$$\lambda =\lambda_{\max}\frac{s_{c}}{K_{S_{c}}+s_{c}}$$

where *s*_*c*_ is the concentration of the growth-limiting carbon source *S*_*c*_, $K_{S_{c}}$ is its Monod saturation or affinity constant and λ_max_ is the maximal growth rate (in batch). We extend this model with thermodynamics by considering the Gibbs free energy potential for the macrochemical equation ([Disp-formula eq11]), obtained from applying ([Disp-formula eq4]) and Hess’s law ([Disp-formula eq7]),

15 $$\Delta \mu_{\mathrm{growth}}=RT\ln\left(\frac{\underset{l}{\prod }p_{l}^{Y_{P_{l}/B}}}{K_{\mathrm{eq}}\underset{k}{\prod }s_{k}^{Y_{S_{k}/B}}}\right)$$

Biomass is also one of the products of the macrochemical equation, but as it has activity equal to one,^40^ its contribution does not appear explicitly in the logarithm. The simplest way to extend the Monod equation with a term that incorporates the displacement from thermodynamic equilibrium is

16 λ=λmaxscKSc+sc(1−∏lplYPl/BKeq∏kskYSk/B)=λmaxscKSc+sc(1−exp(ΔμgrowthRT))

The Δμ_growth_ term is the free energy difference of the macrochemical equation, per C-mole biomass. Just as the original Monod equation, ([Disp-formula eq16]) is a phenomenological equation and should not be interpreted as being mechanistically motivated. It simply accounts for the fact that when substrate and product concentrations in the chemostat medium are at their thermodynamic equilibrium values, the growth rate must be zero. In a real experimental setting, eq 16 is not needed: the measured reactant concentrations and the dilution rate set by the experimenter determine the EPR. Extension ([Disp-formula eq16]) is introduced to allow us to simulate that situation. A similar extension of a specific substrate conversion rate is used by Kleerebezem and Stams.^41^

At steady state, λ = *D*, so the experimenter has control over the growth rate of the culture. The steady-state concentrations of substrates, products and biomass are determined by

17 $$\begin{array}{l} b=\frac{1}{Y_{S_{k}/B}}\left(s_{R,k}-s_{k}\right)=\frac{p_{l}}{Y_{P_{l}/B}}\\ D=\lambda =\lambda_{\max}\frac{s_{c}}{K_{S_{c}}+s_{c}}\left(1-\frac{\underset{l}{\prod }p_{l}^{Y_{P_{l}/B}}}{K_{\mathrm{eq}}\underset{k}{\prod }s_{k}^{Y_{S_{k}/B}}}\right) \end{array}$$

These steady states are thus determined by *D*, and we will use *b*(*D*), *s*_*k*_(*D*), etc., in appropriate places to highlight this dependence. It is easy to see that if any *s*_*k*_ decreases in steady state, then all other substrate concentrations must decrease as well, and all product concentrations must increase. These changes all affect the growth rate by lowering it. Hence, an increase in growth rate can only coincide with an increase in substrate in the medium, and a decrease in product: $s_{k}^{\prime }\left(D\right)>0$ and $p_{l}^{\prime }\left(D\right)<0$ . We also note that *b*′(*D*) < 0.

Usually the concentration of the limiting substrate in the reservoir medium is very high, i.e., $s_{R,c}\gg K_{S_{c}}$ . Under this condition, the maximal dilution rate *D*_max_, above which wash-out of cells is faster than growth, approaches the maximal growth rate λ_max_.^16^ For microbes growing far from equilibrium, irreversibility of the macrochemical equation is approximated via a high *K*_eq_, i.e., a very negative $\Delta \mu_{B,\mathrm{growth}}^{{}^{\circ}}$ .

Lastly, recall (10) for the EPR of a reaction network. Since a chemostat at steady state has fixed substrate, biomass and product concentrations, the Gibbs energy potential may be controlled, and thus compared to the growth rate. For a culture growing at a rate λ = *D* the (temperature-scaled) EPR of the steady-state microbial culture in the chemostat is

18 $$T\Phi \left(D\right)=DX_{\mathrm{growth}}\left(D\right)b\left(D\right)$$

where we used that *X*_growth_ = −Δμ_growth_. We are more interested in the specific (or per-capita) entropy production rate (sEPR) rather than the EPR of the entire culture. The sEPR equals

19 $$\phi \left(D\right)\equiv T\Phi \left(D\right)/b\left(D\right)=DX_{\mathrm{growth}}\left(D\right)$$

This quantity is a better measure for EPR of microbial growth in the chemostat, because the biomass concentration is also a function of growth rate. In order to analyze the EPR at different dilution rates it is therefore better to compute the EPR per unit biomass.

The chemostat model is summarized by eqs 12–16. The main ingredient is a choice of macrochemical equation, which is incorporated into eq 16. We will explore different scenarios, in which either a culture uses a fixed macrochemical equation at different growth rates, or gradually shifts between different ones as a result of changes in protein concentrations as a function of conditions. For these different scenarios we study how the sEPR (eq 19) of the culture behaves as growth rate increases.

## 3 Results

### 3.1 Entropy Production Rate Increases with Rate in Chemical Reaction Networks

The driving force X = −Δμ of a chemical reaction network operating at a fixed temperature and pressure can be increased by increasing substrate concentrations or decreasing product concentrations. From the theory outlined above, we know that i. the rate of a chemical reaction rises with its driving force according to eq 4; ii. the driving force of a network equals a weighted sum of the driving forces of its reactions (eq 9; it is “partitioned” over the reactions); iii. the steady-state flux distribution of a network is a weighted sum of the flux distributions of its EFMs ([Disp-formula eq5]), and each of them is completely determined by a single flux value only. From this, it follows that a chemical reaction network with a fixed macrochemical equation (i.e., fixed weights for its EFMs) described by mass-action kinetics ([Disp-formula eq1]) will always show an increase in EPR ([Disp-formula eq10]) when the driving force of its EFMs rises, because then also all the reaction rates will rise. This can be interpreted as: processes that run at a higher rate also dissipate more energy per unit of time. The details of this argument are given in . The same conclusion holds for a biochemical reaction network with fixed enzyme concentrations.

### 3.2 sEPR May Decrease with Growth Rate in Enzyme-Catalyzed Reaction Networks

When the net driving force of a chemical reaction network with a fixed macrochemical equation is changed, all reactions that had a nonzero rate continue to do so. The relative contribution of the network’s EFMs to the steady-state flux distribution generally depends on the driving force, but EFMs do not appear or disappear by a change in the external concentration of substrates and/or products.

The situation is different in biochemical reaction networks: cells can control the concentration of enzymes by gene expression, and thereby have control which reactions actually run. This control is sometimes independent of the driving force, but it may also influence the driving force directly; by expressing genes that correspond to new reactions, new products may be excreted. This can give rise to a different macrochemical equation, a scenario that can not occur for a chemical reaction network as it lacks enzymes. The network composed out of those new EFMs could therefore have a driving force that is lower than the driving force of the set of EFMs it replaces. This raises the possibility that an increase in growth rate, for instance due to a higher nutrient concentration, may coincide with a *drop* in sEPR when cells start using new EFMs making different products.

This is particularly relevant for microbial cells that shift from metabolism with a high biomass yield at slow growth to a low-yield mode at fast growth. Many microbes that do this show the well-known shift from glucose respiration to overflow metabolism under aerobic conditions.^14^ The high-yield mode of metabolism involves a metabolic network that, for instance, completely combusts glucose into carbon dioxide and water whereas the low-yield mode only degrades glucose partially into overflow byproducts (e.g., acetate, ethanol, formate, lactate, etc.) and carbon dioxide. The low-yield mode of metabolism involves less reactions and generally extracts less free energy from glucose. So, it likely has a lower driving force, yet it is used at a higher growth rate. Such a metabolic shift could therefore be accompanied by a drop in sEPR at increasing growth rate, provided the factor reduction in the driving force is larger than the factor increase of the growth rate. Below we will discuss this mechanism in the context of experimental data, but we will first show the principle with a model of a small metabolic network.

### 3.3 Maximization of Specific Flux Can Lead to Selection of a Pathway with a Lower Driving Force: A Numerical Illustration

Consider the reaction network in Figure 1, which is composed out of seven reversible reactions, each described by enzyme kinetics, and in which the substrate *S* is consumed to produce products *P*_0_, *P*_1_, and/or *P*_2_. Concentrations of these external metabolites are considered fixed. The network has two EFMs, which can attain a steady state by themselves, or by mixing them together. Their respective driving forces are fixed at $X_{1}=\mu_{S}-\mu_{P_{0}}-\mu_{P_{1}}$ and $X_{2}=\mu_{S}-\mu_{P_{0}}-\mu_{P_{2}}$ . At steady state, we have the flux balances

$$v_{1}=v_{2},,v_{3}=v_{4}=v_{5}=v_{6},,v_{2}+v_{6}=v_{7}$$

**Figure 1:**
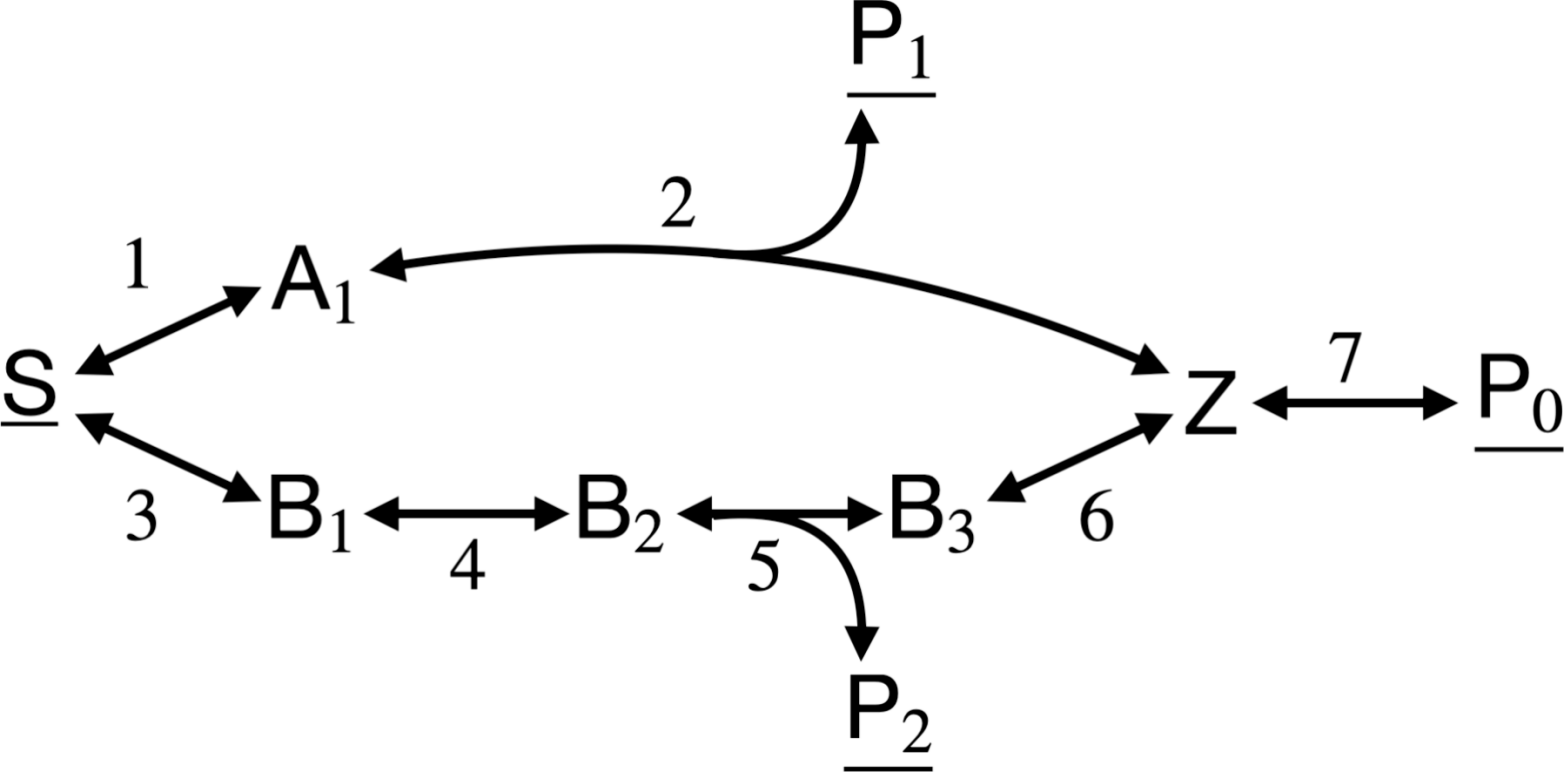
Example metabolic network to illustrate that sEPR may decrease with flux. The concentration of the underlined chemical compounds are considered fixed. EFM 1 is composed out of 3 reactions (1, 2 and 7) and EFM 2 out of 5 reactions (3 to 7). EFM 1 has as thermodynamic driving force $X_{1}=\mu_{S}-\mu_{P_{0}}-\mu_{P_{1}}$ and for EFM 2 the driving force is $X_{2}=\mu_{S}-\mu_{P_{0}}-\mu_{P_{2}}$ .

The flux through EFM 1 is taken to be *v*_1_ and denoted *J*_1_, while for EFM 2 we choose *J*_2_ = *v*_3_. The sEPR of EFM 1 thus equals *X*_1_ *J*_1_ and that of EFM 2 equals *X*_2_ *J*_2_.

First, we consider the two EFMs separately. If the network had been modeled as a chemical reaction network, then *J*_1_ rises with *X*_1_ and *J*_2_ rises with *X*_2_. An increase in the driving force of an EFM thus always leads to an increased sEPR. Their combined sEPR would then also necessarily increase.

Next, we consider the network of Figure 1 as a enzyme-catalyzed reaction network with the product *P*_0_ as a necessary metabolite required for cell growth, and with reaction kinetics specified by enzyme kinetic rate functions. The cell can make *P*_0_ via EFM 1 or 2. Earlier investigation^14^ shows that assuming maximal specific growth rate results in accurate predictions for microbial behavior. High (specific) growth rates require high (specific) reaction rates to support rapid synthesis of cellular components.^42,43^ Since *P*_0_ is required for growth, we therefore assume that the cell chooses the pathway that gives rise to the highest steady-state synthesis flux of *P*_0_, i.e., the rate of reaction 7, per unit of total enzyme concentration invested in the metabolic network. We are therefore considering the maximization of (*J*_1_ + *J*_2_)/*e*_*T*_ with *e*_*T*_ as the sum of the enzyme concentrations of the network. From existing theory,^42,43^ we know that the cell will select just one of the two EFMs.

Which EFM maximizes the specific flux depends on the kinetic properties of the network and the (fixed) external concentrations. Changing the external concentrations might result in a different EFM that attains the highest flux, as is illustrated in Figure 2. It shows the results of a numerical implementation of the reaction network of Figure 1. By tuning kinetic parameters, specifically catalytic rate constants *k*_cat,*j*_, the network can be parametrized such that EFM 2 maximizes the flux for low concentrations of the substrate *S*. Investing the same total enzyme concentration *e*_*T*_ entirely in EFM 1 or EFM 2, the maximal value of *J*_2_ thus exceeds the maximal value of *J*_1_. As *S* increases, the flux-maximizing EFM switches from EFM 2 to EFM 1. Furthermore, by setting appropriate external concentrations we can guarantee that *X*_2_ > *X*_1_ for all values of *S*. So, the switch in the optimal pathway goes together with a drop in the driving force of the network. This behavior can not arise in the chemical reaction networks discussed in the previous section and is a direct result of the extra degrees of freedom in the form of enzyme concentrations a cell has at its disposal.

**Figure 2:**
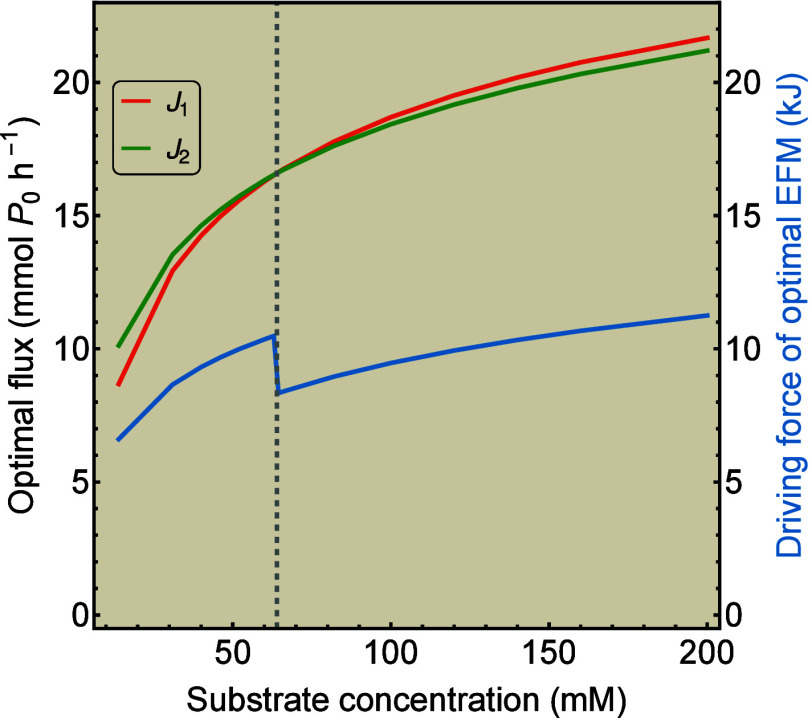
Maximization of specific flux can lead to selection of a pathway with a lower driving force. For both EFMs 1 and 2, its respective optimal fluxes *J*_1_ and *J*_2_ are computed numerically at varying values for the concentration of *S*. At the point where *J*_1_ > *J*_2_, indicated by the gray dashed line, the corresponding driving force of the optimal EFM depicted in blue drops suddenly, as *X*_1_ < *X*_2_.

The effect of this switch on the sEPR of the metabolic network can differ. We may tune the kinetic parameters such that at high *S* concentrations we obtain *J*_1_ > *J*_2_, while *X*_1_ *J*_1_ < *X*_2_ *J*_2_. In this case the decrease in driving force by switching from EFM 2 to EFM 1 overcompensates the increase in flux, resulting in an overall decrease of their product, i.e., the sEPR. This will be analyzed more thoroughly later in this paper. Nevertheless, we can already conclude that maximization of the metabolic rate does not automatically lead to maximization of the sEPR. Generalizing this to the complete metabolic network of the cell implies that sEPR may decrease with growth rate in enzyme-catalyzed reaction networks.

contains general considerations for a thermodynamic consistent and feasible construction of such an example network, together with the explicit conditions for the example network presented here.

### 3.4 A Constant Metabolic Strategy Leads to a Rising Specific Entropy Production Rate as a Function of the Growth Rate in the Chemostat

As explained in the theory section, the chemostat provides a way of studying the (specific) EPR while controlling the growth rate. It allows for the independent measurement of the growth rate (equal to the dilution rate set by the experimenter) and the concentrations of the reactants that appear in the macrochemical equation associated with cell growth. With these ingredients, and the standard Gibbs free energies of formation of the reactants, the driving force ([Disp-formula eq15]) and the sEPR ([Disp-formula eq19]) can be calculated as a function of the growth rate.

When the macrochemical equation is fixed, i.e., when the underlying metabolic network exploits a fixed combination of EFMs, then the driving force and the sEPR both rise with the dilution rate. For instance, when we consider the anaerobic growth of *Saccharomyces cerevisiae* on glucose (C_6_H_12_O_6_), it ferments it to ethanol (C_2_H_6_O) and glycerol (C_3_H_8_O_3_), while forming biomass (CH_1.61_O_0.56_N_0.16_P_0.012_S_0.003_), according to

20 $$1.69C_{6}H_{12}O_{6}+0.16{\mathrm{NH}}_{3}+0.012H_{3}{\mathrm{PO}}_{4}+0.0034H_{2}{\mathrm{SO}}_{4}\to {\mathrm{CH}}_{1.61}O_{0.56}N_{0.16}P_{0.012}S_{0.003}+2.20C_{2}H_{6}O+0.73C_{3}H_{8}O_{3}+2.59{\mathrm{CO}}_{2}+0.11H_{2}O,$$

with ammonium NH_3_ as nitrogen source, H_3_PO_4_ as phosphorus source, and H_2_SO_4_ as sulfur source.^44^ The results of the chemostat model for this macrochemical equation are depicted in Figure 3. Only concentrations of carbohydrates are shown for visual clarity.

**Figure 3:**
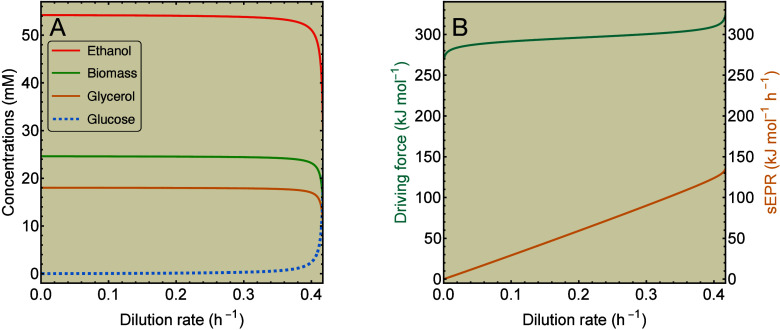
sEPR rises with growth rate for chemostat growth of anaerobic yeast. (A) Concentrations and (B) thermodynamic quantities computed with eqs 15 and 19 for anaerobic chemostat cultivation of *S. cerevisiae* on glucose. Model parameters are given in .

When we consider this fixed macrochemical equation in a chemostat model using steady-state eqs 17, then we show in that the driving force rises with the growth rate. This follows essentially from the way the growth rate ([Disp-formula eq16]) depends on substrates and products, and the fact that substrate concentrations increase with the growth rate, while product concentrations decrease. Hence, the sEPR also rises with the growth rate, as is also illustrated by Figure 3B. We also provide a second example, for the Gram-negative bacterium *Klebsiella aerogenes*, with similar results in . Parameters for both models are given in . A Mathematica implementation of the chemostat model for different organisms can be found in the .

### 3.5 Shifts in Metabolic Strategies Can Lead to a Reduced sEPR as a Function of the Growth Rate in the Chemostat

Many microorganisms gradually shift metabolic strategies as a function of growth rate in aerobic, nutrient-limited chemostats after a critical dilution rate (*D*_*c*_). For instance, *S. cerevisiae* shifts from glucose respiration to fermentation into ethanol^45^ or *L. lactis* shifts from mixed-acid fermentation (formation of acetate, formate and ethanol) to homolactic fermentation (lactate formation).^46^ This implies that the net macrochemical equation of growth is the combination of two such equations (in the absence of maintenance requirements, which are not taken into account in this paper), one for each metabolic strategy, as explained in Section 2.5. The substrate and product yields on biomass are a convex combination of the corresponding quantities of both strategies. Focusing on the shift in *S. cerevisiae*, the yield of metabolite *i* on biomass depends on the dilution rate *D* as

21 $$Y_{i/B}\left(D\right)=\{\begin{array}{ll} Y_{i/B}^{\mathrm{res}}, & D<D_{c}\\ Y_{i/B}^{\mathrm{res}}\left(1-\alpha \left(D\right)\right)+Y_{i/B}^{\mathrm{fer}}\alpha \left(D\right), & D\geq D_{c} \end{array}$$

where $Y_{i/B}^{\mathrm{res}}$ and $Y_{i/B}^{\mathrm{fer}}$ are the yields of metabolite *i* for respiration and fermentation, respectively. Here, we introduce a mixing function α­(*D*), which can be interpreted as the fraction of biosynthetic resources directed to fermentation. So, this mixing function is equivalent to a convex coefficient (see the discussion above eq 8) and is therefore also denoted by α. Experimental data^29,47^ suggests that relations for uptake and excretion rates *q*_*i*/*B*_(*D*) = *DY*_*i*/*B*_(*D*) are affine. In , we show that this implies a mixing function of the form

22 $$\alpha \left(D\right)=\frac{D_{f}\left(D-D_{c}\right)}{D\left(D_{f}-D_{c}\right)},D_{c}\leq D\leq D_{f}$$

The parameter *D*_*f*_ represents the dilution rate at which the organism would have replaced respiration completely by fermentation, and can therefore be called the ‘final’ dilution rate. It should be noted that *D*_*f*_ is only a fitting parameter in our models and is not an observable dilution rate (it generally exceeds the maximal growth rate in the chemostat, because the microbial culture will have washed out before this final dilution rate is reached). The mixing function ([Disp-formula eq22]) attains values between 0 and 1 for *D*_*c*_ ≤ *D* ≤ *D*_*f*_. More information on modeling shifts in metabolism, other possible forms for the mixing function and an extension to mixing three strategies can be found in .

Because the biological[Fn fn3] standard Gibbs energy $\Delta \mu_{B,\mathrm{growth}}^{{}^{\circ}}$ is directly determined by the yields, it depends on *D* as

23 $$\Delta \mu_{B,\mathrm{growth}}^{{}^{\circ}}\left(D\right)=\{\begin{array}{ll} \Delta \mu_{B,\mathrm{res}}^{{}^{\circ}}, & D<D_{c}\\ \Delta \mu_{B,\mathrm{res}}^{{}^{\circ}}\left(1-\alpha \left(D\right)\right)+\Delta \mu_{B,\mathrm{fer}}^{{}^{\circ}}\alpha \left(D\right), & D\geq D_{c} \end{array}$$

Here, $\Delta \mu_{B,\mathrm{res}}^{{}^{\circ}}$ and $\Delta \mu_{B,\mathrm{fer}}^{{}^{\circ}}$ are the biological standard Gibbs energy dissipation of respectively respiration and fermentation, which can be obtained from thermodynamic tables such as given in refs 44 and 48 .

To describe the Gibbs energy potential Δμ_growth_(*D*) including contributions from external concentrations in a similar way, eqs 15 and 23 can be combined. Splitting the logarithmic concentration term in separate contributions for respiration and fermentation results in

24 $$\Delta \mu_{\mathrm{growth}}\left(D\right)=\{\begin{array}{ll} \Delta \mu_{\mathrm{res}}\left(D\right), & D<D_{c}\\ \Delta \mu_{\mathrm{res}}\left(D\right)\left(1-\alpha \left(D\right)\right)+\Delta \mu_{\mathrm{fer}}\left(D\right)\alpha \left(D\right), & D\geq D_{c} \end{array}$$

where Δμ_res_(*D*) and Δμ_fer_(*D*) denote the Gibbs energy potential of respiration and fermentation including contributions from external concentrations, which therefore change with *D*. Saadat et al.^11^ developed a simple model for overflow metabolism that incorporates a similar combination of metabolic strategies and their Gibbs energy potential, however with different extra assumptions.

As the mixing function α­(*D*) describes mixing of metabolic strategies that changes with dilution rate, we have extended the chemostat model from Section 2.6 to microorganisms with a metabolic shift. We have constructed three different models of the yeast *S. cerevisiae* shifting from glucose respiration to fermentation into ethanol. Each of these models uses the chemostat model as basis, but has different macrochemical equations for respiration and fermentation as main ingredients. For model 1, macrochemical equations from Battley^44^ are used as inputs. Battley describes respiratory growth on glucose by *S. cerevisiae* with

$$0.52C_{6}H_{12}O_{6}+0.16{\mathrm{NH}}_{3}+2.12O_{2}+0.012H_{3}{\mathrm{PO}}_{4}+0.0031H_{2}{\mathrm{SO}}_{4}\to {\mathrm{CH}}_{1.61}O_{0.56}N_{0.16}P_{0.012}S_{0.003}+2.13{\mathrm{CO}}_{2}+2.59H_{2}O$$

which has a standard Gibbs energy potential Δμ_*B*,res_^°^ = −1046 kJ/mol. Equation 20 refers to the macrochemical equation from^44^ associated with ethanol fermentation, with Δμ_*B*,fer_^°^ = −357 kJ/mol. Model 2 uses macrochemical equations for respiration and fermentation of yeast from Heijnen and Dijken,^48^ with respective standard Gibbs energy potentials Δμ_*B*,res_^°^ = −467 kJ/mol and Δμ_*B*,fer_^°^ = −255 kJ/mol. Model 3 uses macrochemical equations that we have constructed by fitting a genome-scale model to experimental chemostat data of ref 29. Model 3 contains five different macrochemical equations, as it has two critical growth rates *D*_*c*1_, *D*_*c*2_ and also acetate excretion by another metabolic strategy with Δμ_acefer,1_^°^ = −357 kJ/mol. The standard Gibbs energy potential for respiration is Δμ_res,1_^°^ = −330 kJ/mol and Δμ_ethfer_^°^ = −294 kJ/mol for ethanol fermentation. For *D* > *D*_*c*2_, these values change slightly due to different stoichiometric coefficients in the macrochemical equations. A detailed description of each model including parameters is given in .

From these input macrochemical equations and the specific flux data of ethanol, as measured by,^29^ we can determine the fraction of glucose directed to fermentative growth for each yeast model. The appropriate mixing function for each model is obtained by fitting two different functional forms to these investment fractions. The form ([Disp-formula eq22]) based on linear flux dependencies is used first, unless the fitting parameters *D*_*c*_, *D*_*f*_ have unrealistic values. In that case we use a different form for the mixing function based on hyperbolic flux dependencies, which is described in detail in .

As an example, Figure 4 shows the metabolite yields and the specific fluxes that are calculated with eq 21 for model 3. Its mixing function(s) are obtained from fitting the appropriate convex combination of the corresponding macrochemical equations to the ethanol and acetate fluxes as measured by.^29^ This should result in specific fluxes for model 3 that are comparable with this experimental data. Indeed, the model fits the specific flux data relatively well, except for oxygen.

**Figure 4:**
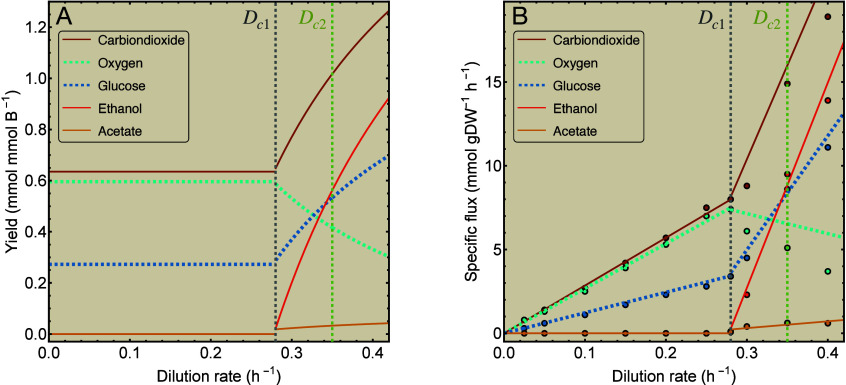
Comparison of model 3 with the data from ref 29. (A) Yields and (B) uptake and excretion rates computed with eq 21 for model 3 of overflowing *S. cerevisiae*. To determine the mixing of strategies for this model, it is fitted to experimental data for acetate and ethanol flux, which are represented by dots. The resulting model fluxes are represented as lines. Substrates are shown by dashed lines, products by solid lines. The vertical dashed lines represent the critical growth rates. Data is reused with permission from ref 29 (Copyright 1992 American Society for Microbiology).

Equations 21–24 are used as ingredients for each yeast model, instead of one macrochemical equation as for the case of a constant metabolic strategy. Together with the steady-state concentrations ([Disp-formula eq17]) that are obtained with the chemostat model ([Disp-formula eq12])–([Disp-formula eq16]), these are all ingredients to determine the sEPR ([Disp-formula eq19]) for each model, as a function of the dilution rate. The results are shown in Figure 5. Concentrations of only the key chemical compounds are depicted, which for all models show similar qualitative behavior. They are approximately constant for *D* < *D*_*c*_ (when the yields remain fixed), but change above the critical dilution rates, when the yields become dependent on the dilution rate. Close to *D*_max_, biomass and other products wash out of the vessel, which explains their concentrations approach zero and the glucose concentration rises to the concentration in the medium that flows into the chemostat.

**Figure 5:**
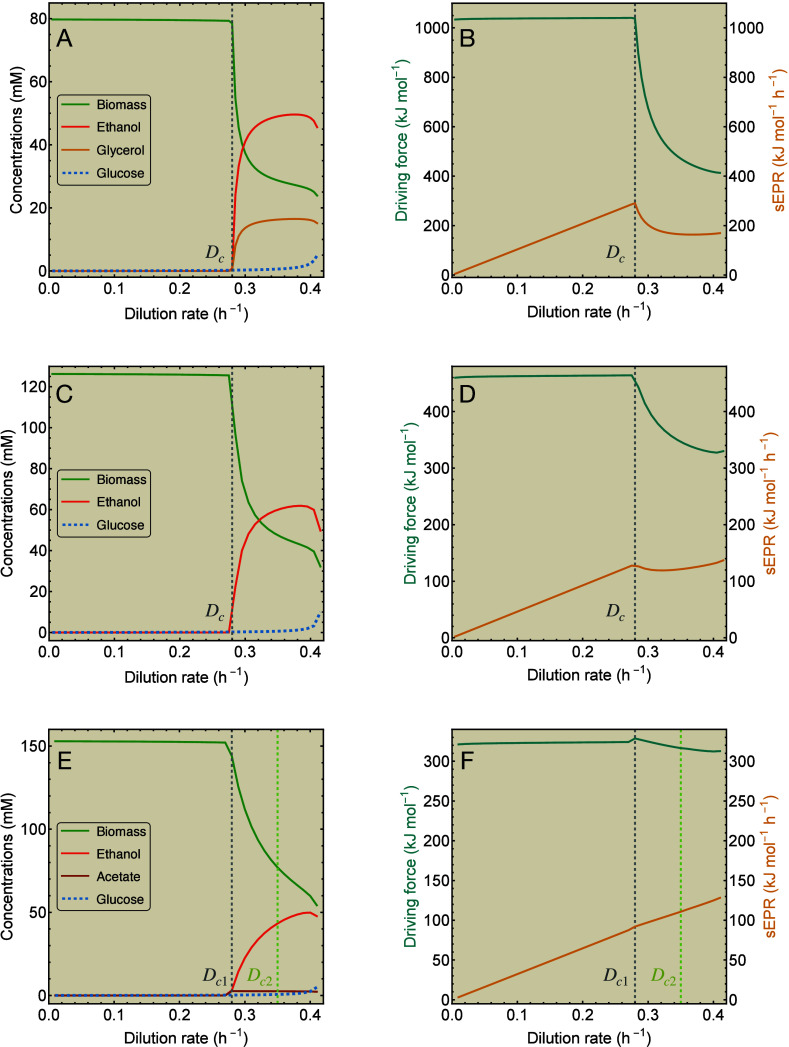
Three chemostat models for overflowing yeast displaying different behaviors of the sEPR. (A) Concentrations and (B) thermodynamic quantities computed with eqs 19 and 24 for model 1 for *S. cerevisiae* growing aerobically on glucose, shifting from respiration to fermentation. (C, D) Same quantities for model 2. (E, F) Same quantities for model 3. Substrates are shown by dashed lines, products by solid lines. The vertical dashed lines represent the critical growth rate(s). Model parameters are given in .

As respiration degrades glucose further than fermentation, the respiratory driving force is generally larger than that of fermentation; *X*_res_ > *X*_fer_. Before cells shift from respiration to fermentation, the driving force remains essentially constant for all models, but decreases after the shift, when *D* > *D*_*c*_. The driving force *X*_growth_(*D*), as depicted in Figure 5, is computed from eq 24 and is thus affected by the ratio of the Gibbs energy potentials Δμ_res_/Δμ_fer_ and the mixing function α­(*D*) for the corresponding model. This results in different behaviors of the sEPR for each model.

The sEPR increases monotonically for *D* < *D*_*c*_, as each yeast model uses only respiration and thus has a fixed macrochemical equation, consistent with Section 3.4. For *D* > *D*_*c*_, the sEPR of model 1 in Figure 5B decreases as a function of the growth rate, while the sEPR of model 2 in Figure 5D only shows a small dip before increasing again. For model 3 in Figure 5F the behavior of the sEPR hardly changes after critical growth. The second shift in this model does not give any observable effect in the calculated quantities.

These results suggest that as growth rate increases, the shift from respiration to fermentation indeed does not need to coincide with an increasing sEPR. In the next section we derive a quantitative rule of thumb that can be used to predict whether the sEPR decreases after the critical growth rate or not.

### 3.6 A Quantitative Criterion for Prediction of Behavior of the sEPR

The previous results suggest that if the driving force decreases faster than linear with the dilution rate *D* after the shift, the sEPR can decrease when *D* ≥ *D*_*c*_. This can happen either when Δμ_res_/Δμ_fer_ is large, or by a steeply increasing mixing function α­(*D*) with *D*.

When growth is far from thermodynamic equilibrium, the concentration contribution to the Gibbs energy change ([Disp-formula eq15]) is negligible with respect to $\Delta \mu_{B,\mathrm{growth}}^{{}^{\circ}}\left(D\right)$ ,^30^ and thus we can approximate this by $\Delta \mu_{\mathrm{growth}}\left(D\right)≃\Delta \mu_{B,\mathrm{growth}}^{{}^{\circ}}\left(D\right)$ . If the mixing function α­(*D*) is of the form ([Disp-formula eq22]), we can then approximate the sEPR by $\phi \left(D\right)≃\phi_{\mathrm{approx}}\left(D\right)=-D\Delta \mu_{B,\mathrm{growth}}^{{}^{\circ}}\left(D\right)$ (see ). During a shift from metabolic strategy 1 to strategy 2 for *D* > *D*_*c*_, a decreasing ϕ_approx_(*D*) with *D* occurs now when the following criterion is met:

$$\phi_{\mathrm{approx}}\left(D_{c}\right)=-D_{c}\Delta \mu_{1}^{o}>-D_{f}\Delta \mu_{2}^{o}=\phi_{\mathrm{approx}}\left(D_{f}\right)$$

i.e., when,

25 $$\frac{\Delta \mu_{1}^{{}^{\circ}}}{\Delta \mu_{2}^{{}^{\circ}}}>\frac{D_{f}}{D_{c}}>1$$

This criterion indicates that a microbe should shift to a strategy with a much smaller driving force, or this shift should occur in a small range of dilution rates to get a decreasing sEPR. presents a complete derivation of this criterion.

The criterion depends on only a few model parameters and is independent of the dilution rate. It can be evaluated for each chemostat model of a microbe displaying a metabolic shift, without explicitly running the complete chemostat model and knowing all other model parameters. The four parameters in ([Disp-formula eq25]) can be plotted as $\left(\frac{D_{f}}{D_{c}},\frac{\Delta \mu_{1}^{{}^{\circ}}}{\Delta \mu_{2}^{{}^{\circ}}}\right)$ . If this coordinate lies above the diagonal given by $\frac{D_{f}}{D_{c}}=\frac{\Delta \mu_{1}^{{}^{\circ}}}{\Delta \mu_{2}^{{}^{\circ}}}$ , then the sEPR is predicted to decrease with growth rate. Figure 6 shows these parameter combinations for the three models discussed in the previous section as blue dots. For model 1 and 2, the sEPR is predicted to decrease, while for model 3 an increase is predicted; these predictions are in line with Figure 5 for model 1 and 3 but not for model 2. provides a detailed description of the criterion prediction for each yeast model.

**Figure 6:**
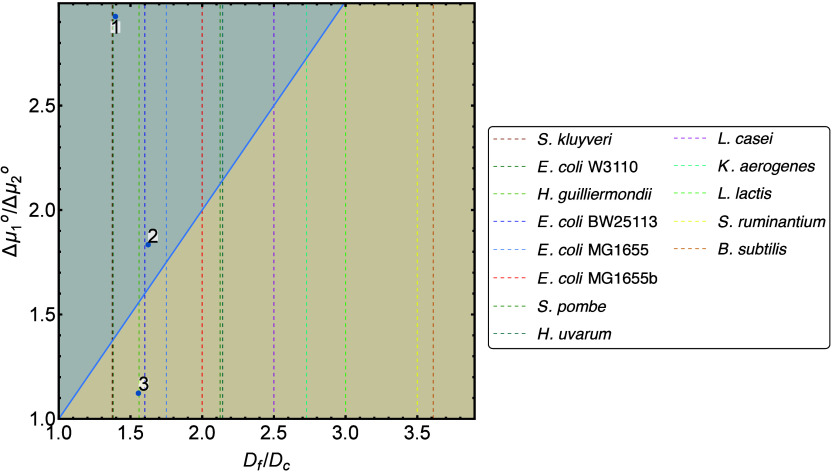
A quantitative criterion can predict the behavior of the sEPR. The sEPR decreases in the blue region. Blue dots represent the three models from this work. Vertical dashed lines depict growth data for metabolic shifts in different other microbes. The growth data is given in .

We have tried to apply the criterion to other organisms that display a metabolic shift. The organisms *L. lactis*, *L. casei*, and *S. ruminantium* shift between strategies during anaerobic growth. The other organisms depicted in Figure 6 show metabolic shifts in aerobic environments, which are similar to overflow metabolism in yeast. Critical and maximal/final dilution rates have been reported for several species, but we generally lack experimental data to compute the ratio of driving forces between the two metabolic strategies. For most of these metabolic shifts, catabolic Gibbs energy potentials are well-characterized for both strategies, but the total Gibbs energies also depend on the anabolic Gibbs energies and the coupling between catabolism and anabolism. These two factors can only be estimated using some assumptions,^30,39,40^ as discussed in , which also results in crude estimations of the total Gibbs energies. However, for the yeast models, we have observed that these values generally have a large impact on the behavior of the sEPR. Thus, evaluating the criterion for these organisms will likely give an unreliable prediction of the sEPR. Therefore, we have plotted these cases using vertical dashed lines in Figure 6.

All available data is presented in . Details about the exact metabolic shift, the corresponding growth data and other information for each organism are described in .

## 4 Discussion and Conclusion

In this work we have studied the relation between specific entropy production rate (sEPR) and growth rate in microbial cells. We have demonstrated that the EPR of a chemical reaction network with a fixed macrochemical equation rises with its reaction rates, because of the direct relation between rate and thermodynamic driving force. Metabolic networks in living cells have more degrees of freedom: they can change enzyme concentrations, allowing them to choose different reactions and alter reaction rates. This may result in decreasing sEPR at faster growth, when the microbe shifts to a metabolic network relying on fewer reactions and meeting a requirement (eq 25).

Studying balanced microbial growth in chemostats allows for controlled analysis of both the growth rate and corresponding sEPR. By extending a chemostat model with thermodynamics we have gained insight in the behavior of the sEPR as a function of growth rate. We have given sufficient conditions for the sEPR to rise with growth rate. For steady-state growth of microorganisms in a chemostat, this is true whenever the net conversion of substrates into products—the macrochemical equation—is fixed.

However, some microbes shift from a high biomass yield to a low-yield strategy at faster growth. Such a metabolic shift changes the net conversion of the metabolic network, as the low-yield strategy only partially degrades the carbon source (e.g., glucose) to e.g., acetate, ethanol or lactate. This partial degradation extracts less free energy and can therefore have a lower driving force at higher growth rates. In general, microbes can compensate for the resulting rate loss through reinvestment of biosynthetic resources into protein expression. A smaller, low-yield pathway can function at higher enzyme concentrations than a larger, high-yield one, by investing the same overall protein concentration in fewer reactions. In this way, the reduction in driving force is compensated by higher rates. This leads to a higher growth rate, but the sEPR does not necessarily increase as well. We further illustrate this using numerical calculations of three different chemostat models for the yeast *S. cerevisiae* that exhibits overflow metabolism at high glucose availability in aerobic environments.

The driving force of respiration in the models differs significantly. It is 2-3-fold higher for model 1 than for model 2 and 3. This is mainly due to differences in stoichiometric coefficients in the macrochemical equations used by these models. This is discussed extensively in . Both models 2 and 3 mix different macrochemical equations, but show very different behavior in their sEPR, even though they have a similar driving force at high growth rates. Model 3 is however based on a genome-scale metabolic network model that uses constraint-based methods based on data and contains a large part of the metabolic network of yeast. One could therefore argue that model 3 gives more reliable results. Ebenhöh et al.^30^ estimate a similar (approximate) linear rise in the catabolic and anabolic sEPR of yeast with increasing dilution rate. However, these findings are not directly comparable to our results for the total sEPR, because of the unspecified energy coupling between catabolism and anabolism. This is discussed further in .

To make more concrete statements, we need additional experimental data confirming our theoretical predictions. There have been calorimetric measurements of heat production in chemostats,^49,50^ but none of these are suited to compare with the results displayed here. We expect this is, next to the difficulty of measuring EPR, because only a few authors consider chemostats as an interesting setup to study thermodynamic properties of living systems. Since experimental evidence is missing and our models show different results, it is too early to state that the sEPR of *S. cerevisiae* will decrease above the critical growth rate. However, the results for all three models show a decreasing driving force as growth rate increases, consistent with others.^30^ Only in specific cases when the decrease in driving force compensates for the increase in growth rate during the shift, this will also result in a decreasing sEPR as growth rate rises. This occurs in one of our chemostat models for overflowing yeast. Every situation in which microbes perform a metabolic shift thus has to be assessed separately, at present.

To give a prediction whether sEPR goes up or down as the growth rate passes a critical value at which a metabolic switch occurs, we have given a simple quantitative rule of thumb, see eq 25. This criterion involves only a small number of measurable parameters: the ratio of critical and final dilution rates, and the ratio of standard Gibbs free energy potentials for the different strategies. However, it turns out that even these parameters have not been experimentally determined for most organisms. To further expose thermodynamic properties of microbes and their metabolic shifts, these parameters should be determined experimentally during chemostat growth. This requires measurements of substrate and product concentrations and fluxes, and calorimetric measurements of heat fluxes. A different method would be to derive macrochemical equations from genome-scale models fitted to chemostat data, as is done in this work.

The thermodynamic chemostat model developed in this work can also be used to study other organisms showing different more exotic shifting behavior in the chemostat. Since it only requires knowledge of macrochemical reactions and a few measurable parameters, it is fairly straightforward to extend. It is therefore an example of the usefulness of the thermodynamic black-box approach, as also highlighted by others.^8,15,51^ This study therefore contributes to recent works^11,12^ that apply these methods to elucidate thermodynamic characteristics of microbial growth. However, to understand what determines the behavior of the (s)­EPR during metabolic shifts, we and others^30^ believe more detailed models of metabolism are required.^12^ This will be investigated in future work. Furthermore, we considered microbes growing far from thermodynamic equilibrium, but there are also cases of growth close to equilibrium,^52^ which might show different behavior of the sEPR. Lastly, our modeling approach opens the door for other thermodynamic analyses of microbes growing in different environments, such as batch conditions. We hope that this work stimulates further research in microbial physiology using thermodynamics, in theoretical as well as experimental directions.

## Supplementary Material





## Footnotes

a Often, $\mu_{C_{i}}$ is called the chemical potential.^18^ We hope not to confuse the reader with this terminology. Also, formation energies are sometimes presented as $\Delta G_{f}^{{}^{\circ}\prime }$ , but as we only look at thermodynamic quantities per mole of a compound, we consistently denote this as μ°′.

b The prime denotes the aqueous environment of the microbes. This notation is suppressed in the rest of this work as we do not consider other environments.

c We hope not to confuse the reader with the 2-fold use of the subscript B, which denotes the biomass for the yields *Y*_*i*/B_, but biological standard conditions for the standard Gibbs energy potentials $\Delta \mu_{B}^{{}^{\circ}}$ .

## Notes

Supplementary Appendices S1–S12: Supplementary text with glossary, general derivations or proofs and extra information on models (PDF)
Source code S13: Example metabolic network. Mathematica code to compute the optimal specific flux of both pathways in the example metabolic network in Figure 1. Used to create Figure 2. Source code S14: Chemostat models for *K. aerogenes* and anaerobic yeast. Mathematica code for models of anaerobic chemostat cultivation of *K. aerogenes* and *S. cerevisiae*. Used to create Figures 3 and . Source code S15: Model 1 for overflowing yeast. Mathematica code for model 1 for chemostat cultivation of *S. cerevisiae* displaying overflow metabolism. Used to create Figures 5A, B. Source code S16: Model 2 for overflowing yeast. Mathematica code for model 2 for chemostat cultivation of *S. cerevisiae* displaying overflow metabolism. Used to create Figures 5C, D. Source code S17: Model 3 for overflowing yeast. Mathematica code for model 3 for chemostat cultivation of *S. cerevisiae* displaying overflow metabolism. Used to create Figures 4 and 5E, F. Data set S18: MCEQs Model 3 from GEM. Excel file with simulation data for chemostat cultivation of *S. cerevisiae* displaying overflow metabolism to construct model 3 from a genome-scale model. Source code S19: Criterion to predict sEPR behavior. Mathematica code for visualization of the quantitative criterion in eq 25 and comparison with data. Used to create Figure 6. Source code S20: Approximation of sEPR. Mathematica code for computing the approximated sEPR for the three models for chemostat cultivation of overflowing *S. cerevisiae*. Used to create Figure S2 (ZIP)
