# Supplementary material for: A Trade-off between Force and Flow May Lead to Reduced Entropy Production Rate during Faster Microbial Growth
Source: J Phys Chem B. 2025 Jun 6;129(24):5923–36. doi: 10.1021/acs.jpcb.4c08559 (PMC12183760; doi:10.1021/acs.jpcb.4c08559)
Supplement: Supplementary file 1 [file jp4c08559_si_001.pdf]

Supplementary Appendices: A Trade-off between Force and Flow may lead to Reduced Entropy Production Rate during Faster Microbial Growth

Maarten J. Droste[1,2], Maaike Remeijer[2], Robert Planqué[1], Frank J. Bruggeman[2]

[1]Department of Mathematics, Amsterdam Center for Dynamics and Computation, Vrije Universiteit Amsterdam, Amsterdam, 1081 HV, the Netherlands
[2]Systems Biology Lab, A-LIFE, AIMMS, Vrije Universiteit Amsterdam, Amsterdam, 1081 HZ, the Netherlands

S1. Glossary

a_i: Conic coefficients for a flux vector decomposition in terms of the EFMs of the reaction network.
b: Biomass concentration in the chemostat vessel.
$c_i^{(e)}$: (Equilibrium) Concentration of chemical compound C_i.
D: Dilution rate of a chemostat.
D_c: Critical growth/dilution rate after which a shift in metabolic strategies occurs.
D_f: Dilution rate at which an organism has replaced its metabolic strategy at slow growth completely by its strategy at fast growth.
D_{max}: Maximal dilution rate in a chemostat, above which wash-out of cells is faster than growth. For $s_{R,c} \gg K_{S_c}$, $D_{max} \simeq \lambda_{max}$.
e_j: Concentration of enzyme j catalysing reaction j.
$\mathbf{E}_i$: Elementary flux mode i of a metabolic network.
e_T: The concentration of all enzymes in a metabolic network summed together.
$f_j(\mathbf{c}')$: Saturation function of enzyme j catalysing reaction j.
$\mathbf{j}$: Vector containing the number of moles of reactants consumed and/or produced in the reactions, obtained from normalising the flux vector $\mathbf{v}$.
$k^{+/-}_{(cat,)j}$: Forward/backward (catalytic) rate constants of reaction j.
$K_{eq,j}$: Equilibrium constant of reaction j, directly related to its standard Gibbs energy dissipation.
K_{S_c}: Monod saturation or affinity constant for carbon source S_c in the chemostat.
$\mathbf{N}$: Stoichiometric matrix of a chemical reaction network with entries n_{ij}.
$\mathbf{N}'$: Stoichiometric matrix extended with stoichiometric coefficients of externally fixed substrate and product concentrations.
p_l: Concentration of product P_l in the chemostat vessel.
$q_{i/B}$: Uptake or excretion rate of compound C_i per mole biomass per hour.
S_c: Carbon source, usually limiting growth.
s_k: Concentration of substrate S_k in the chemostat vessel.
$s_{R,k}$: Concentration of substrate S_k in the reservoir medium.
R: The universal gas constant, 8.314 J/(mol K).
T: Temperature of the system.
v_j: Rate of reaction j.
$v_j^{+/-}$: Forward/backward reaction rates.
X: The thermodynamic driving force of a reaction or pathway per mole, which equals minus the Gibbs free energy potential.
$Y_{i/B}$: Yield in moles of metabolite C_i per mole biomass.
α_i: Convex coefficients when used in macrochemical equations and the entropy production rate.
$\alpha(D)$: Mixing function representing the fraction of resources invested in fermentation.
$\Delta\mu$: The Gibbs free energy potential per mole of a reaction or pathway.
$\Delta\mu^o_{(B)}$: The (biological) standard Gibbs free energy potential per mole of a reaction or pathway.
λ: Growth rate of a microbial culture.
λ_{max}: Maximal growth rate (during batch cultivation).

μ_{C_i}: Gibbs free energy per mole of compound C_i.
$\mu^{o'}_{C_i}$: Standard Gibbs free energy per mole of compound C_i.
Φ: Entropy production rate (EPR).
ϕ: Specific entropy production rate (sEPR) scaled with temperature.
ϕ_{approx}: Approximation of the sEPR that neglects concentration effects.

S2. Considerations for Gibbs energies

For any reaction j, the molar Gibbs free energy potential can be expressed as

$$\begin{aligned} \Delta\mu_j &= \Delta\mu_j^{o'} + RT\ln\left(\frac{\prod_{i=1}^{n_C} c_i^{n_{ij}^-}}{\prod_{i=1}^{n_C} c_i^{n_{ij}^+}}\right) \\ &= RT\ln\left(\frac{\prod_{i=1}^{n_C} c_i^{n_{ij}^-}}{K_{eq,j}\prod_{i=1}^{n_C} c_i^{n_{ij}^+}}\right), \end{aligned} \tag{S1}$$

where the equilibrium constant of the reaction is

$$K_{eq,j} = e^{\frac{-\Delta\mu_j^{o'}}{RT}} = \frac{\prod_{i=1}^{n_C}(c_i^e)^{n_{ij}^+}}{\prod_{i=1}^{n_C}(c_i^e)^{n_{ij}^-}}.$$

This relation implies that the reaction is in thermodynamic equilibrium $\Delta\mu_j = 0$ when all concentrations are equal to their equilibrium concentrations c_i^e, making the Gibbs energy dissipation a measure for the distance from thermodynamic equilibrium. The standard Gibbs energy of reaction $\Delta\mu_j^{o'}$ can therefore be derived directly from this equilibrium condition, as it represents the change in Gibbs free energy occurring at standard conditions.

Standard conditions represent a reference state, denoted by the superscript ^{o}, which has all concentrations at 1 mol/L, a pH of 7, pressure of 1 bar and a temperature of 298.15 K. For biological systems, concentrations of 1 mmol/L are more realistic. These biological standard conditions, which are mostly used in this work, are therefore indicated by an extra subscript $_B$. Note that this changes the reference state and thereby also the standard Gibbs energies (and the corresponding equilibrium constants), but not the values of the Gibbs energies as calculated by eq. (S1). Furthermore, as microbes live in an aqueous environment, all reactions occur in an aqueous solution which changes the corresponding Gibbs energies as well. This is usually denoted by a prime ', but this notation is suppressed in the rest of this work as we only consider aqueous environments. Moreover, the water concentration hardly changes during microbial growth. Therefore, water is not taken into account in calculating Gibbs energy changes, as it remains at its equilibrium concentration.

More information on these different standard conditions and their implications can be found on the eQuilibrator website [1]. This is a useful tool for calculating standard Gibbs energies in different conditions. A complete analysis of the effects of a change in standard conditions on the corresponding $\Delta\mu^o$ is performed by Popovic [2].

To calculate $\Delta\mu_j^o$ for a chemical reaction, the formation energies $\mu^o_{C_i}$ at standard conditions of all reactants need to be known, which can be calculated with methods like component contributions [3]. They are tabulated in different studies of thermodynamics of microbial growth [4; 5; 6]. These formation energies represent the change in Gibbs free energy during the formation reaction of one mole of the compound. Just as the Gibbs energy dissipation $\Delta\mu_j$ can be expressed in terms of molar Gibbs free energies of the reactants, $\Delta\mu_j^o$ can be calculated as

$$\Delta\mu_j^o = \sum_{i=1}^{n_C} n_{ij}\mu^o_{C_i}. \tag{S2}$$

It is common practice to define a reference frame for the formation energies by setting some of them to zero, which can be convenient during calculations or measurements. However, this makes it harder to compare literature values for formation energies, as these are different in each reference frame. This also changes the corresponding Gibbs energies of reaction, which makes it important to be consistent in the use of formation energy tables during modeling.

In the chemostat models constructed in this work, $\Delta\mu^o_{growth}$ is a parameter for each macrochemical equation. For macrochemical equations extracted from the literature the value for $\Delta\mu^o_{growth}$ is given in the corresponding reference. All values are such that growth is always far-from-equilibrium. Recalculation shows that indeed most references use different standard conditions and reference frames, to which we changed our simulations consistently. Details for our models are described in Supplementary Sections S5, S8 and S9.

Because of the difference in standard conditions and reference frames, we performed a check by calculating $\Delta\mu^o_{growth}$ for each model with the table from Heijnen and Kleerebezem [7], which uses the thermodynamic reference frame. This is the most complete table we could find and therefore serves well as a calculation check. The resulting $\Delta\mu^o_{growth}$ for the macrochemical equations from Heijnen and Dijken [4] in model 2 only have a maximum difference of 3%, as their table is almost completely comparable to the table in [7]. For the macrochemical equations from Battley [5] in model 1 the maximum difference in $\Delta\mu^o_{growth}$ was about 19%. This large discrepancy can however be attributed to the difference in standard conditions. For example, Battley uses standard concentrations of 1 mM instead of 1 M. Therefore a fair comparison of $\Delta\mu^o_{growth}$ values calculated with these different tables is actually not possible. Also, it should be noted that these differences in $\Delta\mu^o_{growth}$ do not affect the qualitative behaviour of the sEPR in Figure 5.

S3. Mathematical proof for increase of flux and EPR as function of the driving force in chemical reaction networks

Here we present a mathematically precise version of the argument made in Section 3.1. This argument states that the EPR and flux always increase with thermodynamic driving force in a chemical reaction network with mass action kinetics rate laws, provided that the net conversion of substrates into products remains fixed.

Consider a chemical reaction network at fixed pressure and temperature in a non-equilibrium steady-state. It consists of substrates $\mathbf{S}$, internal metabolites $\mathbf{C}$ and products $\mathbf{P}$ with respective steady-state concentrations $\mathbf{s}, \mathbf{c}, \mathbf{p}$. The conversion

$$\sum_k n_k S_k \to \sum_l n_l P_l \tag{S3}$$

is fixed. The matrix $\mathbf{N}'$ encodes the of stoichiometry the network, while $\mathbf{N}$ is the submatrix of $\mathbf{N}'$ that contains the rows for the internal metabolites only. The network contains r reactions, each reaction j running at rate $v_j(\boldsymbol{\sigma}_j, \boldsymbol{\rho}_j)$ depending on the substrate concentrations $\boldsymbol{\sigma}_j$ and product concentrations $\boldsymbol{\rho}_j$ of the reaction.

We assume that the reactions occur in a well-mixed medium, so that the rates v_j are determined by the law of mass-action and can be written as

$$v_j(\boldsymbol{\sigma}_j, \boldsymbol{\rho}_j) = v_j^+(\boldsymbol{\sigma}_j) - v_j^-(\boldsymbol{\rho}_j),$$

with

$$v_j^+(\boldsymbol{\sigma}_j) = k_j^+ \prod_k \sigma_{k,j}^{n_{k,j}} \text{ and } v_j^-(\boldsymbol{\rho}_j) = k_j^- \prod_l \rho_{l,j}^{n_{l,j}}.$$

The rate constants k_j^+ and k_j^- are assumed to be independent of the state of the system. Now we claim that increasing one or more substrate concentrations s_k or decreasing one or more product concentrations p_l results in both a higher flux v_r through the network, and also a higher EPR.

Proof. As discussed in the main text, the Gibbs energy dissipation corresponding to reaction j is

$$\begin{aligned} \Delta\mu_j &= \sum_l n_{l,j}\mu_{\rho_{l,j}} - \sum_k n_{k,j}\mu_{\sigma_{k,j}} \\ &= RT \ln\left(\frac{\prod_l \rho_{l,j}^{n_{l,j}}}{K_{eq,j}\prod_k \sigma_{k,j}^{n_{k,j}}}\right). \end{aligned} \tag{S4}$$

If the network forms an EFM, it has a flux vector $\mathbf{E}$ that may be normalised such that

$$(\mathbf{S}^T\ \mathbf{C}^T\ \mathbf{P}^T)\mathbf{N}'\mathbf{E} = \sum_k -n_k S_k + \sum_l n_l P_l.$$

The total Gibbs energy dissipation corresponding to the conversion (S3) is then given by

$$\Delta\mu_{tot} = \sum_{j=1}^{r} E_j \Delta\mu_j = RT \ln \left(\frac{\prod_l p_l^{n_l}}{\prod_{j=1}^{r} K_{eq,j} \prod_k s_k^{n_k}} \right). \tag{S5}$$

The corresponding driving forces are now given by $X_j = -\Delta\mu_j$, and $X_{tot} = -\Delta\mu_{tot}$.

As the products of the net conversion are fixed, we may assume that any steady state flux distribution contains a nonzero flux through reaction r (producing one of those products). The EFMs of the network $\{\mathbf{E}_i\}_i$ are normalised at their last entries r, i.e., $E_{i,r} = 1$ for all i. For a steady-state flux vector of this network satisfying $\mathbf{Nv} = \mathbf{0}$ we may thus also introduce $\mathbf{w}$ as the corresponding vector with last element 1, i.e., satisfying

$$\mathbf{v} = (v_1, ..., v_r)^T = v_r(\frac{v_1}{v_r}, ..., 1)^T \equiv v_r(w_1, ..., 1)^T = v_r\mathbf{w}.$$

This vector $\mathbf{w}$ can be written as a convex combination $\mathbf{w} = \sum_{i=1}^{N_{EFMs}} \alpha_i \mathbf{E}_i$ of EFMs with $0 \leq \alpha_i \leq 1$ for all i and $\sum_{i=1}^{N_{EFMs}} \alpha_i = 1$ [8]. This decomposition is non-unique [9]. For the steady-state flux vector $\mathbf{v}$ this yields

$$\mathbf{v} = v_r \sum_{i=1}^{N_{EFMs}} \alpha_i \mathbf{E}_i. \tag{S6}$$

By (S5), each EFM has driving force $X_{EFM,i} = \sum_{j=1}^{r} E_{i,j} X_j$. This implies that the driving force of $\mathbf{v}$ can be written as

$$X_{tot} = \sum_{i=1}^{N_{EFMs}} \alpha_i X_{EFM,i} = \sum_{j=1}^{r} w_j X_j.$$

So the driving force is given by a similar convex sum as the flux vector. The EPR of the chemical reaction network is now given by

$$\begin{aligned} \Phi &= \sum_{j=1}^{r} v_j X_j \\ &= \sum_{j=1}^{r} \sum_{i=1}^{N_{EFMs}} v_r \alpha_i E_{i,j} X_j \\ &= v_r \sum_{i=1}^{N_{EFMs}} \alpha_i \sum_{j=1}^{r} E_{i,j} X_j \\ &= v_r \sum_{i=1}^{N_{EFMs}} \alpha_i X_{EFM,i} \\ &= v_r X_{tot}. \end{aligned} \tag{S7}$$

Suppose now that we increase a substrate concentration s_k or decrease a product concentration (or both). Then by (S5) the driving force of each EFM $X_{EFM,i}$ that converts substrates into products according to (S3) increases, and hence so does X_{tot}. Moreover, since $X_{EFM,i} = \sum_{j=1}^{r} E_{i,j} X_j$, the driving force of at least one reaction j must be higher than before. With the assumed mass action kinetics, this reaction thus has a higher flux than before. But in this EFM, all fluxes have fixed ratios, so all fluxes must have increased. We conclude that v_r has increased, and by (S7) also the EPR. □

It should be noted that this proof assumes that all EFMs of the reaction network have the same net conversion (S3). This condition can be relaxed, if we assume that the driving forces $X_{EFM,i}$ of all EFMs increase, irrespective of the net conversion by that EFM. This could be realized by increasing all substrate concentrations or decreasing all product concentrations. Assuming[4] furthermore that every EFM contains reaction r and thus has nonzero v_r results in a rising EPR. This follows from a similar argument as in the proof above, as all fluxes and all driving forces increase, so both v_r and X_{tot} have increased as well.

[4]This extra assumption is now required; since the conversion can differ per EFM, v_r is not necessarily nonzero in every EFM.

This version of the proof allows for more freedom in the flux vector, as different EFMs can have different conversions. The proof above allows for more freedom in the external concentrations, as only one substrate or product concentrations needs to change to obtain an increasing driving force X_{tot}.

S4. Thermodynamic consistent construction of a toy metabolic network

S4.1. **General considerations for constructing a toy metabolic network.** Small example networks are useful to describe and understand essential properties of metabolic networks, without having to worry about intricate biochemical details. Although this makes the analysis much easier, designing a toy network can be error prone. Kinetic parameters are often chosen freely, while in fact they are restricted by biochemical constraints and the laws of thermodynamics. So, a toy network should be constructed while keeping these rules in mind, a procedure that is sometimes called thermodynamic parameter balancing [10]. Here we summarise the most important considerations for constructing toy metabolic networks.

First of all, the network should satisfy the basic relations as described in Section 2. Internal metabolites are in steady-state, represented by $\dot{\mathbf{c}} = \mathbf{N}\mathbf{v} = \mathbf{0}$. The rate v_j at which an enzyme-catalysed reaction j runs from S to P in the network is given by some enzyme-kinetic relation $v_j = k^+_{cat,j} e_j f_j(s,p)$. For reversible Briggs-Haldane kinetics, which is commonly used, the saturation function is given by

$$f_j(s,p) = k^+_{cat,j} \frac{s/K_s \left(1 - \frac{p}{sK_{eq}}\right)}{1 + s/K_s + p/K_p}. \tag{S8}$$

It is also possible to decompose the rate v_j in a forward and backward rate as in eq (2). However, the convenient property of notation (S8) is that it automatically satisfies the Haldane relation [11], which for this type of kinetics can be written as

$$K_{eq} = \frac{\prod_l (p^e_l)^{n_l}}{\prod_k (s^e_k)^{n_k}} = \frac{k^+_{cat} \prod_l K_{p_l}}{k^-_{cat} \prod_l K_{s_k}}. \tag{S9}$$

This relation implies a constraint on the kinetic parameters and affinities of every reaction, reducing thereby the degrees of freedom. Equation (S8) is written such that the backward catalytic rate constant $k^-_{cat,j}$ is not included, leaving this as free parameter to tune in the model such that the Haldane relation is satisfied.

If the network contains a closed cycle, this has extra implications. As a closed cycle does not result in any net conversion (there are no external concentrations), its Gibbs energy dissipation (i.e., the total driving force) should vanish: $\Delta\mu_{cycle} = \sum_j \Delta\mu_j = 0$. It can be shown that this condition is equivalent to $\prod_j K_{eq,j} = 1$. This constraint on Gibbs energies or equilibrium constants is called Kirchhoff's loop law, analogous to his law for electrical circuits [12]. This condition holds when the network is in steady-state, hence also when it is in thermodynamic equilibrium. In the later case this condition is called detailed balance.

S4.2. **Specific considerations for our branched pathway example.** The general constraints above can be translated to our example network in Figure 1. The values of the parameters used in the numerical implementation of this network to create Figure 2 satisfy all constraints. A Mathematica implementation of the network is given in the Supporting Information.

The network has two EFMs with fluxes J_1, J_2 and corresponding driving forces $X_1 = \mu_S - \mu_{P_0} - \mu_{P_1}$ and $X_2 = \mu_S - \mu_{P_0} - \mu_{P_2}$. As these depend only on external concentrations, a relation between standard molar free energies of the external metabolites and equilibrium constants of the reactions can be derived for each pathway, using eq. (S2). These are given by

$$\mu^o_S - \mu^o_{P_0} - \mu^o_{P_1} = RT \log(K_1 K_2 K_7) \tag{S10}$$

$$\mu^o_S - \mu^o_{P_0} - \mu^o_{P_2} = RT \log(K_3 K_4 K_5 K_6 K_7), \tag{S11}$$

with $K_j = K_{eq,j}$ for notational convenience. Subtracting the first from the second equation gives

$$\mu^o_{P_1} - \mu^o_{P_2} = RT \log\left(\frac{K_3 K_4 K_5 K_6}{K_1 K_2}\right).$$

This relation is of course automatically satisfied whenever the others are, but it does show another possible conversion in this example network. Indeed, due to reversibility of all reactions, P_2 can be converted into

P_1, and vice versa. This pathway contains an internal cycle, but Kirchhoff's loop law does not apply here as the cycle is not closed: there are still external metabolites P_1 and P_2, whose concentrations are fixed [12].

Comparing the driving forces of EFM 1 and 2 yields

$$X_2 - X_1 = \mu_{P_1} - \mu_{P_2} = \mu^o_{P_1} - \mu^o_{P_2} + RT \log\left(\frac{p_1}{p_2}\right). \tag{S12}$$

Mass conservation implies that P_1 and P_2 have the same elemental composition, so they are isomers. If P_1 and P_2 are in fact the same molecule, then $\mu^o_{P_1} = \mu^o_{P_2}$. In this case, both pathways perform the same net conversion, so also the concentrations of P_1 and P_2 should be equal, resulting in the same driving force for both pathways as concentrations of S and P_0 were already fixed. Also, the network then contains a closed cycle $P_2 \rightleftharpoons P_1$, which satisfies Kirchhoff's loop law as its driving force vanishes, or equivalently $\frac{K_3 K_4 K_5 K_6}{K_1 K_2} = 1$. When constructing a toy network these relations are constraints that need to be satisfied by choosing appropriate parameter values.

When constructing a toy network one can prescribe either concentrations and equilibrium constants, or molar free energies and standard Gibbs energies of reactions to satisfy the constraints. As can be seen from the direct relations between these variables the concentration and thermodynamic descriptions are equivalent. Hence, a model in terms of one set of variables and corresponding parameters suffices. This emphasizes that thermodynamics does not encompass any new information regarding modeling.

The branched pathway considered here serves as an example that as function of the nutrient concentration the cell can shift to a pathway that has a higher flux but a lower driving force. To obtain this result, parameters of both pathways can be tuned while satisfying the aforementioned relations and constraints. As EFM 1 has fewer reactions, it has higher (optimal) enzyme concentrations per step so in general also a higher optimal flux when all catalytic rate constants are comparable. Setting the catalytic rate constants of EFM 1 smaller than the rate constants of EFM 2 brings the optimal fluxes through both pathways closer. As explained earlier, this does not violate the Haldane relations because of the remaining degrees of freedom.

Equation (S12) specifies which parameters determine the driving force difference $X_2 - X_1$. This difference should be positive, which occurs when $\underline{p}_1$ is high while $\underline{p}_2$ is low (compared to their equilibrium concentrations). Another way to obtain this is when $\mu^o_{P_1} - \mu^o_{P_2}$ is large, which happens when the equilibrium constants of the reactions in EFM 1 are smaller than the equilibrium constants of the reactions in EFM 2. This can be interpreted as pathway EFM 1 operating closer to thermodynamic equilibrium, and thereby experiencing a larger product inhibition effect. So, the general procedure is to set $K_{eq,j}$ and $k_{cat,j}$ for EFM 1 to low values compared to $K_{eq,j}$ and $k_{cat,j}$ for EFM 2.

It is even possible to find parameters such that $J_1 > J_2$, but $X_1 J_1 < X_2 J_2$. This case is given by the condition

$$\frac{X_2}{X_1} > \frac{J_1}{J_2} > 1. \tag{S13}$$

Note that this condition is equivalent to the criterion presented in eq. (25).

S5. Notes on chemostat modeling & models for two anaerobic microbes

Here we give a detailed description of the models of two cultures of microorganisms growing anaerobically on glucose. The chemostat model equations are given in Section 2.6. A chemostat volume of $V = 1$ liter is used, such that the flow rate F equals the dilution rate $D = F/V = F$. For each organism, its macrochemical equation is the main model ingredient. Here we detail the choices of these equations for the different scenarios discussed in the text.

According to [5], yeast *Saccharomyces cerevisiae* ferments glucose to ethanol and glycerol as

$$\begin{aligned}&1.69\, C_6H_{12}O_6 + 0.16\, NH_3 + 0.012\, H_3PO_4 + 0.0034\, H_2SO_4\\ &\longrightarrow CH_{1.61}O_{0.56}N_{0.16}P_{0.012}S_{0.003} + 2.20\, C_2H_6O + 0.73\, C_3H_8O_3 + 2.59\, CO_2 + 0.11\, H_2O.\end{aligned}$$

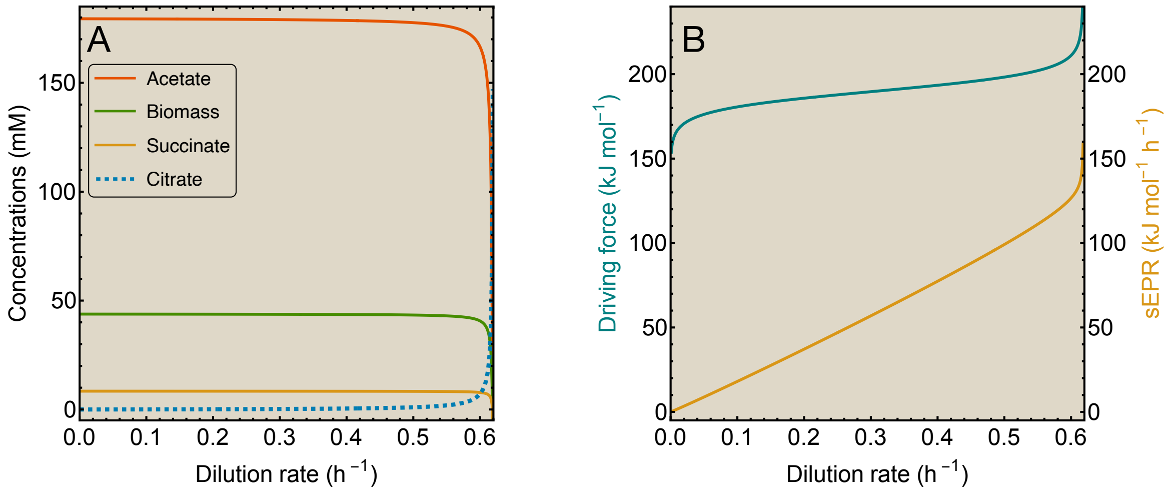

FIGURE S1. **Results of the chemostat model for *K. aerogenes*.** A) Concentrations and B) Thermodynamic quantities for anaerobic growth of *K. aerogenes* on citrate. Substrates are shown by dashed lines, products by solid lines. Model parameters are given in Table S1.

The bacterium *Klebsiella aerogenes* grows anaerobically on citrate, fermenting this to acetate, succinate and formate via [4]

$$2.283\, C_6H_5O_7^{3-} + 4.6\, H_2O + 0.2\, NH_4^+$$
$$\longrightarrow CH_{1.8}O_{0.5}N_{0.2} + 4.096\, C_2H_3O_2^- + 0.192\, C_4H_4O_4^{2-} + 0.068\, CHO_2^- + 3.657\, HCO_3^- + 1.56\, H^+ + 0.63\, H_2.$$

The first product in the macrochemical equation is the biomass, written down in its elemental composition and normalised per C-mole. Most experimental data report the biomass per gram dry weight, which changes the yield units accordingly. Here we adopt the convention of reporting the biomass in moles, to check elemental balance more easily. All model parameters differ per organism and are given in Table S1. The standard Gibbs energies of the macrochemical equations $\Delta\mu^o_{B,growth}$ are calculated using the same standard conditions, reference point and thermodynamic tables as in the work stating the particular macrochemical equation. Note that all substrate concentrations in the reservoir medium are chosen such that only the carbon source is limiting for growth. All other substrates are therefore in excess. Also, the concentration of the carbon source in the reservoir medium $s_{R,c}$ is chosen such that it is much larger than its affinity constant K_{S_c}.

The macrochemical equations and parameters given in Table S1 are all the ingredients required to implement and numerically solve the chemostat model for these organisms in Mathematica. The numerical solution is calculated by rewriting eq. (17) for the steady-state concentrations and the dilution rate in terms of biomass b, and solving this equation for b for $0 \leq D \leq \lambda_{max}$ with a stepsize of 0.001-0.015 h^{-1} (differs per model). The values $b(D)$ are then substituted in the other equations to find all other concentrations and thereby also the driving force and the sEPR.

The concentrations and thermodynamic quantities for *Klebsiella aerogenes* are depicted in Figure S1 and are qualitatively similar to the results for yeast in Figure 3. Again, only concentrations of carbohydrates are shown for visual clarity, but other concentrations are included in the models as well and depend similarly on D. Concentrations and therefore driving force change only very close to the maximal dilution rate $D_{max} \simeq \lambda_{max}$, because growth of these organisms is far from equilibrium, which is included in the models through a high $K_{eq} \gg 1$. In this regime, the C-source approaches its reservoir concentration while products and biomass approach zero due to wash out. Because of this behaviour, X_{growth} and the sEPR indeed increases monotonically in D, for both organisms. Close to the maximal dilution rate, they approach infinity.

Mathematical modeling of the chemostat has a long history [13; 14; 15]. Our model assumes the chemostat contains a well-mixed liquid with only one microbial species, for which sticking to the walls of the bioreactor is negligible. Obviously, our models are not complete. Microbes invest some resources into maintenance, which decreases the observed yields [16]. We do not think this has a large impact on our results. As wash out occurs for growth rates close to the maximal dilution rate D_{max}, the model gives unrealistic results for both concentrations and thermodynamics in the chemostat close to D_{max}; either they approach zero or infinity.

S6. sEPR always increases with growth rate for a fixed metabolic strategy in the chemostat

In general metabolic networks as considered in this work, the cell controls enzyme concentrations which determine the flux and the driving force. The example network in Section 3.3 illustrated that this can result in selection of a pathway with a higher specific flux but a lower driving force. When the macrochemical equation is fixed, this is not possible. We therefore claim that both the driving force and the sEPR rise with growth rate in chemostat conditions.

Proof. To see this, the dependence of the growth rate on the total driving force needs to be analysed. First, we study the steady-state solution of the chemostat model given by eq. (17). For any substrate S_k, its steady-state concentration $s_k(D)$ decreases with increasing biomass concentration $b(D)$. Similarly, for any product P_l, its steady-state concentration $p_l(D)$ increases with $b(D)$. Rewriting the dilution rate D in eq. (17) as function of b now gives that $D'(b) > 0$, as both the Monod relation and the contribution from thermodynamics decrease in b. Reversing this statement now yields that $b'(D) < 0$, which then implies that $s'_k(D) > 0$ and $p'_l(D) < 0$. Inspecting then the subsequent relation (15) between the driving force and the dilution rate then shows that $X'_{growth}(D) > 0$. This shows that, for organisms in a chemostat modelled by a single fixed macrochemical equation, the driving force and therefore the sEPR rises with the growth rate. □

The relations between steady-state concentrations, driving force and the dilution rate are of course assumptions in the chemostat model, but they follow from observed behaviour and fundamental microbiology, such as the Monod relation. Furthermore, the growth rate in eq. (17) is exactly the same as the rate equation for mass-action kinetics in a chemical reaction network, except for the Monod term. So, the argument here follows a similar logic as for chemical reaction networks presented earlier, and therefore has the same conclusions.

S7. Modeling overflow metabolism with a mixing function

The models for overflowing *S. cerevisiae* are similar to the models presented previously. The only difference is that, after a critical dilution rate D_c, the respirative and fermentative macrochemical equations are mixed. This is due to the appearance of active intracellular constraints, which limits the microorganism in its behaviour and requires it to use other strategies to maintain its growth rate [17]. So, constraints are now required to model these microbes accurately, which was not necessary before for the other organisms. Since these constraints do not appear naturally in our chemostat model, the mixing of strategies has to be included explicitly by introducing a mixing function $\alpha(D)$, which is defined as the fraction of resources invested in the fermentation strategy and is equivalent to a convex coefficient in an EFM decomposition of a steady-state flux vector (S6). von Stockar and Birou [18] have developed a similar method to model mixing of strategies in yeast.

Experimental data [19; 20] suggests that uptake and excretion fluxes $q(D)$ in a chemostat are affine in D, i.e., $q(D) = DY(D) = \nu + \tau D$ for some $\nu, \tau \geq 0$. Denoting the mixing function by $\alpha_{lin}(D)$ for this case, the flux $q(D)$ can be expressed as

$$\begin{aligned} q(D) &= \nu + \tau D \\ &= DY_{res}(1 - \alpha_{lin}(D)) + DY_{fer}\alpha_{lin}(D), \end{aligned} \tag{S14}$$

where the second form describes the change in the flux due a changing yield $Y(D)$ as in eq. (21), which is affected by increasing investment in fermentation and decreasing investment in respiration. Solving this for

$\alpha_{lin}(D)$ yields

$$\alpha_{lin}(D) = \frac{\nu + (\tau - Y_{res})D}{(Y_{fer} - Y_{res})D}. \tag{S15}$$

Parameter D_c is determined by the start of the metabolic shift, at which $\alpha_{lin}(D_c) = 0$, which gives $\nu = (Y_{res} - \tau)D_c$. At the 'final' dilution rate $D = D_f$, yeast is using only fermentation, which translates into $\alpha(D_f) = 1$ implying $\nu = (Y_{fer} - \tau)D_f$. Plugging in these two requirements gives the mixing function in the form (22)

$$\alpha_{lin}(D) = \frac{D_f(D - D_c)}{D(D_f - D_c)}. \tag{S16}$$

Note that D_f can not always be attained by the shifting organism. For example, yeast growing at maximal rate in aerobic batch conditions still uses some oxygen [21], so it seems to never reach the growth rate $\lambda = D_f$.

To determine the parameters in the mixing function for each yeast model, it has to be fitted to data. Since the macrochemical equations from Battley [5] for model 1 and Heijnen and Dijken [4] for model 2 do not include accompanying chemostat data, the data from van Hoek et al. [20] is used. This contains flux data at different dilution rates for all important substrates and products of yeast. If the data and macrochemical equations are normalised to the same substrate (in this case glucose), the ethanol flux can be used to determine the fraction of resources invested in fermentation to achieve this flux at dilution rate D. This fraction should be equal to $\alpha_{lin}(D)$. So, the mixing function can be fitted to this mixing data by determining these fractions for different dilution rates and thereby obtaining values for the parameters D_c and D_f.

Performing these fits for our three yeast models can however result in a value for $D_c < 0.28$ h^{-1}, while experiments show that $D_c = 0.28$ h^{-1}. Furthermore, some fits also have a value for $D_f < 0.4$ h^{-1}, while the data shows there is still some oxygen consumed at this dilution rate [20; 21]. This would also imply that yeast has completely shifted to fermentation before growing at its maximal rate. Because of these potential issues, a different functional form for the mixing function is attempted. Based on the seemingly hyperbolic behaviour of the mixing data, we chose a hyperbolic form

$$\alpha_{sat}(D) = \frac{D - D_c}{D - A}, \tag{S17}$$

where A is a different fitting parameter. This function saturates slower to 1 than (S16), as $\lim_{D\to\infty} \alpha_{sat}(D) = 1$. Note that this function still satisfies $\alpha_{sat}(D_c) = 0$. A disadvantage is that this choice results in nonlinear fluxes, while experimental data suggest these to be affine.

To choose either form (S16) or (S17), one would generally compare their goodness of fit to determine which form fits the data best. However, the data from [20] is so sparse that R^2 values are extremely high (> 0.95). So, this is no reliable measure for comparison, as many different forms for $\alpha(D)$ with two fitting parameters could fit the obtained mixing data well. Therefore, we base the choice of the form for the mixing function on the values of the fitting parameters D_c and D_f. We use $\alpha_{lin}(D)$ as default, unless after fitting this to the mixing data we obtain a $D_c < 0.28$ h^{-1} or $D_f < 0.4$ h^{-1}. In that case, we use $\alpha_{sat}(D)$. Based on these criteria a mixing function is determined for each model, which are specified in Supplementary Sections S8 and S9.

Note that the behaviour of the sEPR depends on the choice for the form of the mixing function. Just as the form (S16) results in affine fluxes, it also results in an affine sEPR as function of D, while eq. (S17) yields hyperbolic behaviour. So, the behaviour of the sEPR is therefore not uniquely determined, which makes it hard to draw strong conclusions. Nevertheless, we believe our models gives a description of the sEPR that is as accurate as possible for a theoretical analysis.

S8. Chemostat models for overflowing *S. cerevisiae* based on literature

Models 1 and 2 for overflowing yeast growing in a glucose-limited chemostat are based on macrochemical equations obtained from the literature. Model 1 uses macrochemical equations for respiration and fermentation of *S. cerevisiae* from [5], which are respectively given by

$$\begin{aligned}&0.52\,C_6H_{12}O_6 + 0.16\,NH_3 + 2.12\,O_2 + 0.012\,H_3PO_4\\&+ 0.0031\,H_2SO_4 \longrightarrow CH_{1.61}O_{0.56}N_{0.16}P_{0.012}S_{0.003} + 2.13\,CO_2 + 2.59\,H_2O,\\&1.69\,C_6H_{12}O_6 + 0.16\,NH_3 + 0.012\,H_3PO_4 + 0.0034\,H_2SO_4\\&\longrightarrow CH_{1.61}O_{0.56}N_{0.16}P_{0.012}S_{0.003} + 2.20\,C_2H_6O + 0.73\,C_3H_8O_3 + 2.59\,CO_2 + 0.11\,H_2O.\end{aligned}$$

Based on the criteria as described in Supplementary Section S7, the mixing function used for this model for overflowing yeast is given by

$$\alpha_1(D) = \frac{D - 0.28}{D - 0.26}. \tag{S18}$$

Model 2 uses macrochemical equations for respiration and fermentation of *S. cerevisiae* from Heijnen and Dijken [4], which are respectively given by

$$\begin{aligned}&0.33\,C_6H_{12}O_6 + 0.2\,NH_4^+ + 0.93\,O_2\\&\longrightarrow CH_{1.8}O_{0.5}N_{0.2} + 0.98\,HCO_3^- + 0.4\,H_2O + 1.18\,H^+,\\&1.1904\,C_6H_{12}O_6 + 0.2\,NH_4^+ + 1.63\,H_2O\\&\longrightarrow CH_{1.8}O_{0.5}N_{0.2} + 2.0308\,C_2H_6O + 2.081\,HCO_3^- + 2.28\,H^+.\end{aligned}$$

The mixing function used for this model for overflowing yeast is given by

$$\alpha_2(D) = \frac{D - 0.28}{D - 0.24}. \tag{S19}$$

Both references use slightly different biomass compositions, and differ significantly in stoichiometric coefficients for glucose and ethanol (for fermentation). Also, oxygen and carbon-dioxide appear in some macrochemical equations, which are gases that are not completely dissolved in water. Therefore, their contribution to the concentration term in eq. (15) should only include the dissolved fraction. However, as all growth processes considered here are far from equilibrium, this effect on the total driving force for growth is negligible. It can therefore be assumed that oxygen and carbon-dioxide are completely dissolved in the bioreactor.

All other parameters of these models can be found in Table S1. The standard Gibbs energies are calculated using the thermodynamic tables given in the same reference as we obtained the corresponding the macrochemical equation from. It should be noted that Battley uses biological standard conditions, while Heijnen & van Dijken use non-biological standard conditions. Both use the thermodynamic reference frame. The considerable differences in stoichiometric coefficients between the macrochemical equations of both models cause a discrepancy between the corresponding Gibbs energy dissipations. This is especially the case for respiration, that has a two-told higher Gibbs energy dissipation for model 1 than for model 2.

S9. Chemostat model for overflowing *S. cerevisiae* based on data

Model 3 for overflowing yeast is directly based on the data from [20], whom measured uptake rates and effluxes of different metabolites during growth of *S. cerevisiae* in a glucose-limited chemostat. Macrochemical equations based on this data were reconstructed using the genome-scale model (GEM) Yeast8 [22]. Uptake rates and effluxes for all metabolites and trace elements were obtained with the GEM at different dilution rates $0 < D < 0.4$ h^{-1}, by lower bounds on ethanol and acetate efflux that correspond to the measured fluxes of these overflow metabolites at a certain dilution rate D, which was then also the lower bound of the growth rate. The constraint on the non-growth associated maintenance reaction was removed. Under these constraints the glucose uptake rate was minimised. This results in a list of uptake rates and effluxes for all constituents that satisfies elemental balance at each dilution rate. For overflowing yeast at $D > D_{c1}$ these fluxes are a mix of different macrochemical equations (EFMs). An EFM enumerator [23] was used via CNApy [24] to determine the EFMs that were used by the GEM at these dilution rates. For $D > D_{c1}$ the GEM makes use of three EFMs to satisfy the three constraints for biomass, ethanol and

acetate flux, which can be characterised as respiration, ethanol fermentation and acetate fermentation. The elemental composition of biomass was computed from the elemental balance condition. The GEM shows an inexplicable overconsumption of phosphate ions and overproduction of diphosphate, which seems to be a model incoherence and is therefore neglected. This results however in an oxygen unbalance, explaining the low coefficient for oxygen in the biomass composition.

The macrochemical equation for respiration, which is used both below and above D_{c1} is given by

$$\begin{aligned} &0.273\,\mathrm{C_6H_{12}O_6} + 0.165\,\mathrm{NH_4^+} + 0.60\,\mathrm{O_2} + 0.0117\,\mathrm{H^+} \\ &\longrightarrow \mathrm{CH_{1.62}O_{0.395}N_{0.165}} + 0.635\,\mathrm{CO_2} + 1.16\,\mathrm{H_2O}. \end{aligned}$$

The macrochemical equation for ethanol fermentation is given by

$$\begin{aligned} &0.714\,\mathrm{C_6H_{12}O_6} + 0.165\,\mathrm{NH_4^+} + 0.286\,\mathrm{O_2} + 0.0117\,\mathrm{H^+} \\ &\longrightarrow \mathrm{CH_{1.62}O_{0.395}N_{0.165}} + 0.988\,\mathrm{C_2H_6O} + 1.31\,\mathrm{CO_2} + 0.852\,\mathrm{H_2O}. \end{aligned}$$

The macrochemical equation for acetate fermentation is given by

$$\begin{aligned} &0.305\,\mathrm{C_6H_{12}O_6} + 0.165\,\mathrm{NH_4^+} + 0.584\,\mathrm{O_2} + 0.0117\,\mathrm{H^+} \\ &\longrightarrow \mathrm{CH_{1.62}O_{0.395}N_{0.165}} + 0.104\,\mathrm{C_2H_3O_2} + 0.624\,\mathrm{CO_2} + 1.25\,\mathrm{H_2O}. \end{aligned}$$

Note that all strategies make use of oxygen, which is energetically favorable and available in the medium of the GEM. Mixing coefficients, which describe the fraction of resources directed towards a strategy, were determined for each EFM by finding a unique flux and computing the fraction of glucose investment to attain this flux. Equivalently, we have computed the fraction of biomass synthesized by this EFM, as all EFMs produce biomass. This fraction is more convenient as the biomass yield is normalised at every D. The intuitive choice for the unique fluxes is the ethanol efflux for ethanol fermentation and acetate efflux for acetate fermentation. However, during the analysis it turned out that around $D = 0.35\ \mathrm{h}^{-1}$, the sum of the mixing coefficients of these two fermentation strategies would exceed 1, which indicates that the cell would need to use more glucose than available to satisfy the constraints. As this is not possible, this behaviour hints at a second critical growth rate $D_{c2} = 0.35\ \mathrm{h}^{-1}$, after which new strategies emerge with different yields that can satisfy the constraints. This is in line with other recent findings using genome scale modeling of yeast [21]. After this second shift at $D > D_{c2}$, it turns out that both macrochemical equations for respiration and acetate fermentation change. The yields in the macrochemical equations change slightly and they start to produce ethanol as well. The ethanol fermentation strategy remains the same. This model therefore includes five macrochemical equations, three of which are given above. For $D > D_{c2}$, respiration is given by

$$\begin{aligned} &0.339\,\mathrm{C_6H_{12}O_6} + 0.165\,\mathrm{NH_4^+} + 0.547\,\mathrm{O_2} + 0.0117\,\mathrm{H^+} \\ &\longrightarrow \mathrm{CH_{1.62}O_{0.395}N_{0.165}} + 0.739\,\mathrm{CO_2} + 1.11\,\mathrm{H_2O} + 0.154\,\mathrm{C_2H_6O}. \end{aligned}$$

The macrochemical equation for acetate fermentation for $D > D_{c2}$ is given by

$$\begin{aligned} &0.656\,\mathrm{C_6H_{12}O_6} + 0.165\,\mathrm{NH_4^+} + 0.339\,\mathrm{O_2} + 0.0117\,\mathrm{H^+} \\ &\longrightarrow \mathrm{CH_{1.62}O_{0.395}N_{0.165}} + 0.104\,\mathrm{C_2H_3O_2} + 1.16\,\mathrm{CO_2} + 1.01\,\mathrm{H_2O} + 0.788\,\mathrm{C_2H_6O}. \end{aligned}$$

For these five macrochemical equations the corresponding standard Gibbs energies were computed using the table from Heijnen and Dijken [4], as this was the most complete table for these reactants. To find the formation energy for biomass, its elemental composition was compared with an extensive list in [2] containing formation energies for different microorganisms. The biomass sharing closest resemblance with our biomass composition has a formation energy of $\mu_B^o = -72.69$ kJ/mol. The resulting values are given in Table S1, next to other parameters for model 3. A control calculation with the table from [7] showed only small deviation of at most 3%, indicating that our method gives reasonable values for the standard Gibbs energies of macrochemical equations. It should be noted that the standard Gibbs energy for acetate fermentation is even more negative than for respiration, which contradicts the idea that respiration reduces glucose further and can therefore extract more free energy. However, looking at the macrochemical equations of respiration and acetate fermentation in both critical regimes, one can observe that acetate fermentation has more in common with respiration than actual (ethanol) fermentation. This can be seen from the high biomass yield on glucose, low carbondioxide efflux and low oxygen uptake rate. In general, the standard Gibbs energies

for respiration for model 1 and 2 are much more negative than the value found for model 3. To investigate these two interesting features, more detailed data would be required than is currently provided by [20].

As there are two critical regimes, this requires four mixing functions (one for each fermentation strategy in each regime) to fit to the mixing coefficients. However, the data by [20] only includes 4 measurements for $D > D_{c1} = 0.28$ h^{-1}, which is too sparse to properly fit different forms of the mixing function, as also explained in Supplementary Section S7. To make a better choice for the mixing function, the existing data is extended using a linear interpolation. With this interpolation the ethanol and acetate efflux for each 0.28 h^{-1} $\leq D \leq 0.40$ h^{-1} with a stepsize of 0.1 were determined and used as constraints in the GEM. Running the GEM now yields fluxes, macrochemical equations and thereby mixing coefficients at more values of the dilution rate than in the original data. It should be noted that this gave inconclusive results on the exact moment of the second shift, as multiple values for D_{c2} were compatible with a sum of mixing coefficients below 1. Based on [21] we have fixed the second shift at $D_{c2} = 0.35$ h^{-1}. To summarise, the yields $Y_{i/B}(D)$ for this model of overflowing yeast in each growth regime are now given by

$$\text{(S20)}\quad Y_{i/B}(D) = \begin{cases} Y^{res1}_{i/B} & D < D_{c1} \\ Y^{res1}_{i/B}(1 - \alpha_{eth1}(D) - \alpha_{ace1}(D)) + Y^{eth}_{i/B}\alpha_{eth1}(D) + Y^{ace1}_{i/B}\alpha_{ace1}(D) & D_{c1} \leq D \leq D_{c2} \\ Y^{res2}_{i/B}(1 - \alpha_{eth2}(D) - \alpha_{ace2}(D)) + Y^{eth}_{i/B}\alpha_{eth2}(D) + Y^{ace2}_{i/B}\alpha_{ace2}(D) & D_{c2} < D \leq \lambda_{max}. \end{cases}$$

Similar equations can be written down for the (standard) Gibbs energies, just as in eq.(23). The four mixing functions for each fermentation strategy in each regime are given by

$$\begin{aligned} \alpha_{eth1}(D) &= \frac{2.76(D - 0.28)}{D} \\ \alpha_{ace1}(D) &= \frac{0.86(D - 0.22)}{D} \\ \alpha_{eth2}(D) &= \frac{2.43(D - 0.31)}{D} \\ \alpha_{ace2}(D) &= \frac{0.86(D - 0.22)}{D}. \end{aligned}$$

The mixing function for the acetate fermentation strategy is the same in both critical regimes. This is because only this EFM produces acetate, for which the efflux increases linearly in D according to the data. As this model contains two shifts and five EFMs, most fitting parameters in these four mixing functions lose their biological interpretation. Therefore, we use the default form (S16) for all mixing functions in this model.

Figure 4B shows the rates $q_{i/B}(D) = DY_{i/B}(D)$ calculated with eq. (S20) together with the data from van Hoek et al. for the most important reactants. This shows that model 3 describes the data reasonably well, except for the oxygen uptake rate. This is probably due to unrealistic behaviour of the GEM. The results of the chemostat model for this description of overflowing yeast in terms of five EFMs are depicted in Figures 5E, 5F.

S10. Derivation of quantitative criterion for behaviour of the sEPR

In Section 3.6 we have used a quantitative criterion involving an approximation of the sEPR to predict the behaviour of the sEPR for $D > D_c$ for our three yeast models and other shifting organisms. Here we will present a derivation of this criterion.

For $D > D_c$ the sEPR decreases if $\phi'(D) < 0$. This derivative can be computed directly using the (mixed) yields and standard Gibbs energies (21). However, as $\Delta\mu_{growth}(D)$ is nonlinear and includes all substrates and products of the macrochemical equation, the result will not be insightful. Therefore, some assumptions are required to find a simple and clear criterion. The Gibbs energy dissipation can be written in terms of a standard Gibbs energy plus a concentration effect

$$\text{(S21)}\qquad \Delta\mu_{growth}(D) = \Delta\mu^o_{B,growth}(D) + RT\ln\left(\frac{\prod_l p_l(D)^{Y_{P_l/B}}}{\prod_k s_k(D)^{Y_{S_k/B}}}\right),$$

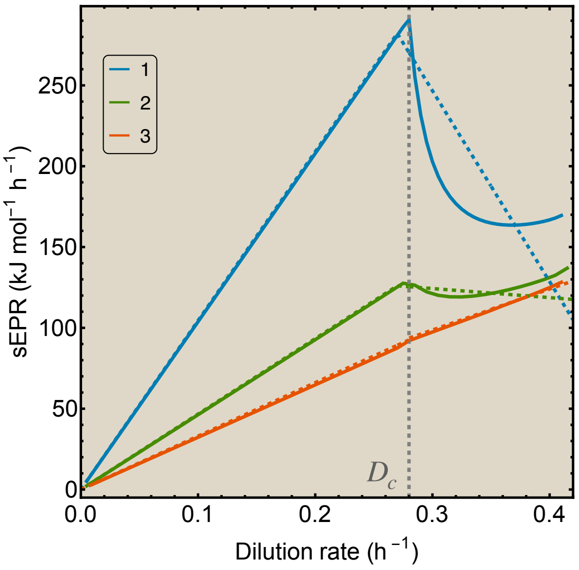

FIGURE S2. **Approximation of the sEPR for the three chemostat models for overflowing yeast.** Approximation is depicted as a dashed line. For model 1 and 3 it captures the characteristic behaviour, but not for model 2. The vertical dashed line represents the critical growth rate $D_c = 0.28$ h^{-1}.

by applying $K_{eq}(D) = \exp(-\Delta\mu^o_{B,growth}(D)/RT)$. The dependence of $\Delta\mu^o_{B,growth}$ on the dilution rate follows from mixing of two metabolic strategies 1 and 2 as

$$\Delta\mu^o_{B,growth}(D) = \Delta\mu^o_1(1-\alpha(D)) + \Delta\mu^o_2\alpha(D),\ D \geq D_c, \tag{S22}$$

similar to eq. (23). Growth of all organisms considered in this work is far from thermodynamic equilibrium, as $\Delta\mu^o_{B,growth} < -100$ kJ/mol for all macrochemical equations. This means that the concentration effect is negligible for the Gibbs energy dissipation, so $\Delta\mu_{growth}(D) \simeq \Delta\mu^o_{B,growth}(D)$. To derive a simple criterion, we assume that $\alpha(D)$ has the form (22) resulting in affine fluxes $q_j(D)$. Together, these assumptions result in an sEPR that is also approximately affine for $D \geq D_c$, as

$$\begin{aligned}\phi(D) &\simeq \phi_{approx}(D)\\ &= -D\Delta\mu^o_{B,growth}(D)\\ &= -D\Delta\mu^o_1 + (\Delta\mu^o_1 - \Delta\mu^o_2)\frac{D_f(D-D_c)}{D_f - D_c}\\ &= D\left(\frac{\Delta\mu^o_1 D_c - \Delta\mu^o_2 D_f}{D_f - D_c}\right) - \frac{\Delta\mu^o_1 - \Delta\mu^o_2}{D_f - D_c}D_f D_c.\end{aligned} \tag{S23}$$

Hence, the sEPR decreases for $D > D_c$ when

$$\frac{\Delta\mu^o_1 D_c - \Delta\mu^0_2 D_f}{D_f - D_c} < 0, \quad D_f > D_c,$$

which may be summarised as

$$\frac{\Delta\mu^o_1}{\Delta\mu^o_2} > \frac{D_f}{D_c} > 1. \tag{S24}$$

Each microbial species that displays a metabolic shift is characterised by a coordinate $(\frac{D_f}{D_c}, \frac{\Delta\mu^o_1}{\Delta\mu^o_2})$. If this coordinate lies above the (dimensionless) diagonal given by $\frac{\Delta\mu^o_1}{\Delta\mu^o_2} = \frac{D_f}{D_c}$ (the blue region in Figure 6), then the sEPR is predicted to decrease.

S11. Data for different organisms with a metabolic shift to predict their sEPR behaviour

This section contains information on growth and standard Gibbs energy data obtained for organisms that are represented in Figure 6. As briefly explained in Section 3.6, most of these organisms are represented by vertical dashed lines. The reasons for this will be elaborated on here.

S11.1. **Data for yeast models.** For the three yeast models constructed in this work, coordinates $\left(\frac{D_f}{D_c}, \frac{\Delta\mu_1^o}{\Delta\mu_2^o}\right)$ were determined and represented in Figure 6 as blue dots to predict the behaviour of their sEPR. For model 1 and 2 it should be noted that these use a mixing function of the form (S17), which includes no parameter D_f. In order to evaluate the criterion for these models, we compute D_f from mixing functions of the form (22) that were not used in the corresponding chemostat model, as otherwise the criterion can not be evaluated for these models. It should also be noted that D_f is ambiguously defined for organisms mixing more than two strategies, as happens for model 3. In that case we have calculated D_f as the dilution rate at which there would be only ethanol fermentation, as also the standard Gibbs energy of ethanol fermentation was used for $\Delta\mu_2^o$.

Based on the coordinates found for the three yeast models, the prediction by the criterion is correct for models 1 and 3, but incorrect for model 2. Such a false prediction could follow from the approximation $\phi(D) \simeq \phi_{approx}(D)$. To check the reliability of this approximation, we have plotted $\phi(D)$ and $\phi_{approx}(D)$ for each yeast model in Figure S2. One can conclude from this that $\phi_{approx}(D)$ is indeed a crude approximation for models 1 and 3, but it does capture the qualitative behaviour of $\phi(D)$.

For model 2 the criterion predicted a decreasing sEPR, which is indeed the behaviour of $\phi_{approx}(D)$. However, for this model it turns out that approximating the sEPR loses the key qualitative behaviour of $\phi(D)$, as this first shows a drop before rising again. As the criterion is independent of D, it can not predict such non-monotonic behaviour.

Nevertheless, we conclude that the criterion (S24) is useful to predict behaviour of the (approximated) sEPR, as it depends only on a few model parameters. It therefore allows for a simple characterisation of thermodynamics of organisms with metabolic shifts.

S11.2. **Data for other organisms with metabolic shifts.** All organisms except *L. lactis*, *L. casei* and *S. ruminantium* show metabolic shifts in aerobic environments, from respiration at low growth rates to fermentation at high growth rates. Hence, these shifts are similar to overflow metabolism in yeast. The reason that these organisms are depicted by continuous vertical dashed lines in Figure 6 instead of one point is because not all required data for evaluating the criterion (25) is available. For these organisms, data on critical growth rates D_c when fermentation is initiated is usually available. D_f is not measurable for most organisms, either because of chemostat limitations which decrease $D_{max} < D_f$ or because, like yeast, the organism still respires a small fraction of its glucose uptake at maximal growth rate λ_{max}. Instead, λ_{max} can be used as a lower bound on D_f, as it is the maximal growth rate measured in batch at rich medium, which also gives a lower bound for D_f/D_c. This is a fair approximation to assess the criterion, as we are interested to find organisms that could potentially show a decreasing sEPR, which is more likely for lower D_f. So, if $\lambda_{max}/D_c \gg 1$, this indicates a high probability of increasing sEPR, as the criterion for decreasing sEPR requires $\frac{\Delta\mu_1^o}{\Delta\mu_2^o} > \frac{D_f}{D_c} > \frac{\lambda_{max}}{D_c} > 1$.

A problem arises when examining the standard Gibbs energies of the two strategies that the organisms use during the shift. As we have observed for yeast, the standard energy $\Delta\mu_1^o$ of respiration is usually available, but the standard energy $\Delta\mu_2^o$ of the exact fermentation strategy used by the organism is harder to retrieve, for reasons explained in Supplementary Section S9 and S12. Therefore, the ratio $\frac{\Delta\mu_1^o}{\Delta\mu_2^o}$ can not be calculated unambiguously for these organisms, which translates to an unknown y-coordinate in Figure 6 and consequently vertical dashed lines for these aerobic metabolic shifts.

Table S2 contains the growth rate data for the organisms with metabolic shifts during aerobic growth, which determines their x-coordinate in Figure 6. For *S. kluyveri* [25] and *H. uvarum* [26] it must be noted that even though the authors observed a metabolic shift, they still classified the yeast as being Crabtree-negative. Also the maximal growth rate of these yeasts is unknown, so an estimate for λ_{max} is obtained by extrapolating data for *S. kluyveri* and by taking the maximal experimental dilution rate for *H. uvarum*. *K. aerogenes* exhibits overflow metabolism in phosphate-limited chemostats [27]. For *E. coli* W3110, $D_f \gg 0.8$ h^{-1}, as oxygen uptake seems to remain constant for this strain [28]. For *E. coli* MG1655 the critical and

maximal growth rates were measured by two different studies [29; 30], finding different results, so both are included.

The organisms *L. lactis*, *L. casei* and *S. ruminantium* show metabolic shifts during anaerobic growth. Data for their critical growth rates D_c and λ_{max} are also contained in Table S2. *L. lactis* shifts from heterolactic fermentation at low dilution rates to homolactic fermentation at high dilution rates. The critical dilution rate is however ill-defined, as lactate is already produced at the onset of measuring [31]. A similar shift is observed in *L. casei* [32] and *S. ruminantium* [33]. These anaerobic organisms are also represented by vertical dashed lines in Figure 6, for reasons explained in the following section.

S12. General catabolic shifts and methods to approximate Gibbs energy dissipations for growth

For the aforementioned and other metabolic shifts, catabolic Gibbs energy dissipations are generally well-characterised for both strategies. These shifts also occur in the catabolic part of the growth process, but the (total) Gibbs energy dissipation is given by [34]

$$\Delta\mu_{growth} = f_{cat}\Delta\mu_{cat} + \Delta\mu_{ana}, \tag{S25}$$

where f_{cat} represents the number of times the catabolic reaction runs to generate all the energy (ATP) required to synthesize one mole biomass, if both processes are properly normalised. Hence, $\Delta\mu_{growth}$ is also determined by anabolism and the coupling of these processes. As anabolism corresponds (mostly) to biosynthesis, it usually costs energy, so therefore $\Delta\mu_{ana} \geq 0$ in most cases [7]. Due to coupling through ATP, anabolism is driven by catabolism. We have tried to circumvent these unknown parameters by comparing the ratios $\frac{\Delta\mu^o_{growth,1}}{\Delta\mu^o_{growth,2}}$ and $\frac{\Delta\mu^o_{cat,1}}{\Delta\mu^o_{cat,2}}$ for two general metabolic strategies $1, 2$. If the second ratio is larger than the first, we can use the criterion by substituting the ratio of the catabolic Gibbs energy potentials for the total Gibbs energy potentials of growth. There are indications that these ratios are comparable, for example the observed positive relation between the ATP yield and $\Delta\mu_{cat}$ [7]. However, because no strict bounds exist on both $\Delta\mu_{ana}$ and f_{cat}, the required inequality can not be proven.

So, in order to compute the Gibbs energy potentials of both strategies that are exploited during the shift, which are required to evaluate the criterion (S24), the values for $\Delta\mu_{ana}$ and f_{cat} need to be calculated for an organism exploiting such a shift. In principle, it is possible to estimate an anabolic reaction and the corresponding Gibbs energy change. Heijnen and Kleerebezem [7] and Ebenhöh et al. [35] describe similar methods to obtain a general anabolic reaction that only depends on the carbon source, biomass composition and their degree of reduction. Kleerebezem and van Loosdrecht [34] use redox half reactions to get a better estimate for the anabolic reaction. However, both methods assume that every organism with similar biomass composition growing on glucose can be represented by the same anabolic reaction.

The factor f_{cat} is directly related to the yield of biomass on the carbon source Y_{B/S_c} [34]. This yield is usually experimentally determined for a single growth strategy. However, when two strategies are mixed for $D > D_c$, also the yield is a function of the growth rate. It is therefore hard to determine Y_{B/S_c} for the second strategy, without constructing a complete chemostat model and determining a mixing function for such an organism. This implies that $f_{cat,2}$ for strategy 2 can not be determined in most cases, or can only be estimated roughly. Another way to obtain f_{cat} for both strategies is by assuming steady-state of the ATP concentration in the cell. Then, $f_{cat} = \frac{m_{ana}}{m_{cat}}$, with m_{ana} the ATP demand by anabolism and m_{cat} the ATP yield of the employed catabolic mode. However, in practice, anabolism also produces ATP during synthesis of biosynthetic precursors, such as pyruvate and α-ketoglutarate. These precursors are synthesized during glycolysis and in the TCA cycle, which are generally interpreted as (solely) catabolic pathways. Obtaining the fraction of the ATP synthesis that is anabolic requires detailed knowledge of the metabolic pathways of the organism of interest. This makes it hard to determine the factor f_{cat}, and thereby also hard to determine $\Delta\mu_{ana}$.

In conclusion, the methods mentioned here can only give crude estimations of $\Delta\mu_{ana}$, Y_{B/S_c} and f_{cat}, and hence also a crude estimation of the complete macrochemical equation and its Gibbs energy potential. During this study it was observed that yields and (standard) Gibbs energy potentials in the macrochemical equations have a large impact on the behaviour of the sEPR. Therefore, we consider a prediction for these organisms based on the criterion (S24) and these crude estimations to be unreliable. Therefore, as thermodynamic data is incomplete, also these shifting organisms are depicted as vertical dashed lines in Figure 6.

**Organism**	**Environment**	λ_{max} (h^{-1})	**Reservoir concentrations** (mM)	K_{S_c} (mM)	$\Delta\mu^o_{growth}$ (kJ/mol)	D_c (h^{-1})
K. aerogenes [4]	Anaerobic	0.62 [36]	$[C_6H_5O_7^{3-}]_R = 100$ $[NH_4^+]_R = 100$	0.24 [37]	-185 [4]	-
S. cerevisiae [5]	Anaerobic	0.42 [20]	$[C_6H_{12}O_6]_R = 41.7$ [20], $[NH_3]_R = 50$, $[H_3PO_4]_R = 50$, $[H_2SO_4]_R = 50$	0.12 [38]	-357 [5]	-
S. cerevisiae (Model 1) [5]	Aerobic	0.42	$[C_6H_{12}O_6]_R = 41.7$ $[O_2]_R = 250$ $[NH_3]_R = 50$ $[H_3PO_4]_R = 50$ $[H_2SO_4]_R = 50$	0.12	$\Delta\mu^o_{B,res} = -1046$, $\Delta\mu^o_{B,fer} = -357$	0.28
S. cerevisiae (Model 2) [4]	Aerobic	0.42	$[C_6H_{12}O_6]_R = 41.7$ $[O_2]_R = 250$ $[NH_4^+]_R = 50$	0.12	$\Delta\mu^o_{res} = -467$, $\Delta\mu^o_{fer} = -255$	0.28
S. cerevisiae (Model 3)	Aerobic	0.42	$[C_6H_{12}O_6]_R = 41.7$ $[O_2]_R = 250$ $[NH_4^+]_R = 50$ $[H]_R = 50$	0.12	$\Delta\mu^o_{res,1} = -330$ $\Delta\mu^o_{ethfer} = -294$ $\Delta\mu^o_{acefer,1} = -357$ $\Delta\mu^o_{res,2} = -326$ $\Delta\mu^o_{acefer,2} = -329$	$D_{c1} = 0.28$ $D_{c2} = 0.35$

TABLE S1. **Table with parameter values for each organism for which a chemostat model has been developed in this work.** The references next to the organism names are the reference from which its macrochemical equation is taken. The last three cases of *S. cerevisiae* are simulated with a model including overflow metabolism. Concentrations in the reservoir medium are chosen such that only the carbon source, which is the first substrate in the list, is limiting growth. For parameters with the same value for different yeast models that are taken from a reference, only the first mention of the parameter value contains the corresponding reference.

**Organism**	**Environment**	D_c (h^{-1})	λ_{max} (h^{-1})	**Reference**
S. pombe	Aerobic	0.16	0.34	[39]
B. subtilis	Aerobic	0.36	1.3	[40]
H. uvarum	Aerobic	0.28	0.6	[26]
H. guilliermondii	Aerobic	0.25	0.39	[41]
S. kluyveri	Aerobic	0.51	0.7	[25]
K. aerogenes	Aerobic	0.22	0.6	[27]
E. coli W3110	Aerobic	0.58	0.8	[28]
E. coli MG1655	Aerobic	0.4	0.7	[29]
E. coli MG1655b	Aerobic	0.25	0.5	[30]
E. coli BW25113	Aerobic	0.5	0.8	[42]
L. lactis	Anaerobic	0.15	0.6	[31]
L. casei	Anaerobic	0.2	0.5	[32]
S. ruminantium	Anaerobic	0.2	0.7	[33]

Table S2. **Table with growth data for organisms displaying a shift in metabolic strategies.**

References

[1] Avi Flamholz, Elad Noor, Arren Bar-Even, and Ron Milo. eQuilibrator—the biochemical thermodynamics calculator. *Nucleic Acids Research*, 40(D1):D770–D775, 11 2011. ISSN 0305-1048. doi: 10.1093/nar/gkr874. URL `https://doi.org/10.1093/nar/gkr874`.

[2] Marko Popovic. Thermodynamic properties of microorganisms: determination and analysis of enthalpy, entropy, and Gibbs free energy of biomass, cells and colonies of 32 microorganism species. *Heliyon*, 5(6):e01950, 2019. ISSN 2405-8440. doi: https://doi.org/10.1016/j.heliyon.2019.e01950. URL `https://www.sciencedirect.com/science/article/pii/S2405844018385852`.

[3] Elad Noor, Hulda Haraldsdóttir, Ron Milo, and Ronan Fleming. Consistent estimation of Gibbs energy using component contributions. *PLoS computational biology*, 9:e1003098, 07 2013. doi: 10.1371/journal.pcbi.1003098.

[4] J. J. Heijnen and J. P. Van Dijken. In search of a thermodynamic description of biomass yields for the chemotrophic growth of microorganisms. *Biotechnology and Bioengineering*, 39, 1992.

[5] Edwin Battley. A theoretical study of the thermodynamics of microbial growth using *Saccharomyces cerevisiae* and a different free energy equation. *The Quarterly review of biology*, 88:69–96, 06 2013. doi: 10.1086/670529.

[6] Robert A. Alberty. Calculation of standard transformed Gibbs energies and standard transformed enthalpies of biochemical reactants. *Archives of Biochemistry and Biophysics*, 353 (1):116–130, 1998. ISSN 0003-9861. doi: https://doi.org/10.1006/abbi.1998.0638. URL `https://www.sciencedirect.com/science/article/pii/S0003986198906385`.

[7] Joseph J. Heijnen and Robbert Kleerebezem. *Bioenergetics of Microbial Growth*, pages 1–66. John Wiley and Sons, Ltd, 2010. ISBN 9780470054581. doi: https://doi.org/10.1002/9780470054581.eib084. URL `https://onlinelibrary.wiley.com/doi/abs/10.1002/9780470054581.eib084`.

[8] Julien Gagneur and Steffen Klamt. Computation of elementary modes: a unifying framework and the new binary approach. *BMC bioinformatics*, 5:1–21, 2004.

[9] Jean-Marc Schwartz and Minoru Kanehisa. Quantitative elementary mode analysis of metabolic pathways: the example of yeast glycolysis. *BMC Bioinformatics*, 7(1):186, 2006. doi: 10.1186/1471-2105-7-186. URL `https://doi.org/10.1186/1471-2105-7-186`.

[10] Timo Lubitz, Marvin Schulz, Edda Klipp, and Wolfram Liebermeister. Parameter balancing in kinetic models of cell metabolism. *The Journal of Physical Chemistry B*, 114(49):16298–16303, 12 2010. doi: 10.1021/jp108764b. URL `https://doi.org/10.1021/jp108764b`.

[11] J.B.S. Haldane. *Enzymes.* Monographs on Biochemistry. Longmans, Green, 1930. URL `https://books.google.nl/books?id=ECnyUM5WJQgC`.

[12] Hong Qian, Daniel A. Beard, and Shou-dan Liang. Stoichiometric network theory for nonequilibrium biochemical systems. *European Journal of Biochemistry*, 270 (3):415–421, 2003. doi: https://doi.org/10.1046/j.1432-1033.2003.03357.x. URL `https://febs.onlinelibrary.wiley.com/doi/abs/10.1046/j.1432-1033.2003.03357.x`.

[13] Hal L. Smith and Paul Waltman. *The Theory of the Chemostat: Dynamics of Microbial Competition.* Cambridge Studies in Mathematical Biology. Cambridge University Press, 1995. doi: 10.1017/CBO9780511530043.

[14] Patrick De Leenheer, Simon A. Levin, Eduardo D. Sontag, and Christopher A. Klausmeier. Global stability in a chemostat with multiple nutrients. *Journal of Mathematical Biology*, 52(4):419–438, 2006. doi: 10.1007/s00285-005-0344-4. URL `https://doi.org/10.1007/s00285-005-0344-4`.

[15] J. Gijs Kuenen. *Continuous Cultures (Chemostats)*, pages 743–761. Academic Press, Oxford, fourth edition edition, 2019. ISBN 978-0-12-811737-8. doi: https://doi.org/10.1016/B978-0-12-801238-3.02490-9. URL `https://www.sciencedirect.com/science/article/pii/B9780128012383024909`.

[16] S. J. Pirt and Cyril Norman Hinshelwood. The maintenance energy of bacteria in growing cultures. *Proceedings of the Royal Society of London. Series B. Biological Sciences*, 163(991):224–231, 1965. doi: 10.1098/rspb.1965.0069. URL `https://royalsocietypublishing.org/doi/abs/10.1098/rspb.1965.0069`.

[17] Daan de Groot, Julia Lischke, Riccardo Muolo, Robert Planqué, Frank Bruggeman, and Bas Teusink. The common message of constraint-based optimization approaches: overflow metabolism is caused by

two growth-limiting constraints. *Cellular and Molecular Life Sciences*, 77, 02 2020. doi: 10.1007/s00018-019-03380-2.

[18] U. von Stockar and B. Birou. The heat generated by yeast cultures with a mixed metabolism in the transition between respiration and fermentation. *Biotechnology and Bioengineering*, 34(1):86–101, 1989. doi: https://doi.org/10.1002/bit.260340112. URL `https://onlinelibrary.wiley.com/doi/abs/10.1002/bit.260340112`.

[19] Markus Basan, Sheng Hui, Hiroyuki Okano, Zhongge Zhang, Yang Shen, James R. Williamson, and Terence Hwa. Overflow metabolism in *Escherichia coli* results from efficient proteome allocation. *Nature*, 528(7580):99–104, 2015. doi: 10.1038/nature15765. URL `https://doi.org/10.1038/nature15765`.

[20] Pim van Hoek, J.P. van Dijken, and Jack Pronk. Growth rate on fermentative capacity of baker's yeast. *Applied and environmental microbiology*, 64:4226–33, 11 1998. doi: 10.1128/AEM.64.11.4226-4233.1998.

[21] Ibrahim E Elsemman, Angelica Rodriguez Prado, Pranas Grigaitis, Manuel Garcia Albornoz, Victoria Harman, Stephen W Holman, Johan van Heerden, Frank J Bruggeman, Mark MM Bisschops, Nikolaus Sonnenschein, et al. Whole-cell modeling in yeast predicts compartment-specific proteome constraints that drive metabolic strategies. *Nature communications*, 13(1):801, 2022.

[22] Hongzhong Lu, Feiran Li, Benjamín J. Sánchez, Zhengming Zhu, Gang Li, Iván Domenzain, Simonas Marcišauskas, Petre Mihail Anton, Dimitra Lappa, Christian Lieven, et al. A consensus *S. cerevisiae* metabolic model Yeast8 and its ecosystem for comprehensively probing cellular metabolism. *Nature Communications*, 10(1):3586, 2019. doi: 10.1038/s41467-019-11581-3. URL `https://doi.org/10.1038/s41467-019-11581-3`.

[23] Marco Terze. *Large scale methods to enumerate extreme rays and elementary modes.* PhD thesis, ETH Zurich, 2009.

[24] Sven Thiele, Axel von Kamp, Pavlos Stephanos Bekiaris, Philipp Schneider, and Steffen Klamt. CNApy: a CellNetAnalyzer GUI in Python for analyzing and designing metabolic networks. *Bioinformatics*, 38(5):1467–1469, 12 2021. ISSN 1367-4803. doi: 10.1093/bioinformatics/btab828. URL `https://doi.org/10.1093/bioinformatics/btab828`.

[25] Kasper Møller, Christoffer Bro, Jure Piškur, Jens Nielsen, and Lisbeth Olsson. Steady-state and transient-state analyses of aerobic fermentation in *Saccharomyces kluyveri*. *FEMS Yeast Research*, 2(2):233–244, 05 2002. ISSN 1567-1356. doi: 10.1111/j.1567-1364.2002.tb00088.x. URL `https://doi.org/10.1111/j.1567-1364.2002.tb00088.x`.

[26] C. Venturin, H. Boze, G. Moulin, and P. Galzy. Glucose metabolism, enzymic analysis and product formation in chemostat culture of *Hanseniaspora uvarum*. *Yeast*, 11(4):327–336, 1995. doi: https://doi.org/10.1002/yea.320110405. URL `https://onlinelibrary.wiley.com/doi/abs/10.1002/yea.320110405`.

[27] O. M. Neijssel and D. W. Tempest. Bioenergetic aspects of aerobic growth of *Klebsiella aerogenes* NCTC 418 in carbon-limited and carbon-sufficient chemostat culture. *Archives of Microbiology*, 107(2): 215–221, 1976. doi: 10.1007/BF00446843. URL `https://doi.org/10.1007/BF00446843`.

[28] Amit H. Varma and Bernhard O. Palsson. Stoichiometric flux balance models quantitatively predict growth and metabolic by-product secretion in wild-type *Escherichia coli* W3110. *Applied and Environmental Microbiology*, 60:3724 – 3731, 1994. URL `https://api.semanticscholar.org/CorpusID:40496290`.

[29] Annik Nanchen, Alexander Schicker, and Uwe Sauer. Nonlinear dependency of intracellular fluxes on growth rate in miniaturized continuous cultures of *Escherichia coli*. *Applied and Environmental Microbiology*, 72(2):1164–1172, 2006. doi: 10.1128/AEM.72.2.1164-1172.2006. URL `https://journals.asm.org/doi/abs/10.1128/aem.72.2.1164-1172.2006`.

[30] Kaspar Valgepea, Kaarel Adamberg, Ranno Nahku, Petri-Jaan Lahtvee, Liisa Arike, and Raivo Vilu. Systems biology approach reveals that overflow metabolism of acetate in *Escherichia coli* is triggered by carbon catabolite repression of acetyl-CoA synthetase. *BMC Systems Biology*, 4(1):166, 2010. doi: 10.1186/1752-0509-4-166. URL `https://doi.org/10.1186/1752-0509-4-166`.

[31] Anisha Goel, Thomas H. Eckhardt, Pranav Puri, Anne de Jong, Filipe Branco dos Santos, Martin Giera, Fabrizia Fusetti, Willem M. de Vos, Jan Kok, Bert Poolman, et al. Protein costs do not explain evolution of metabolic strategies and regulation of ribosomal content: does protein investment explain an anaerobic bacterial Crabtree effect? *Molecular Microbiology*, 97(1):77–92, 2015. doi: https://doi.org/10.1111/mmi.13012. URL https://onlinelibrary.wiley.com/doi/abs/10.1111/mmi.13012.

[32] Wytske De Vries, Willemina M. C. Kapteijn, E. G. Van Der Beek, and A. H. Stouthamer. Molar growth yields and fermentation balances of *Lactobacillus casei* L3 in batch cultures and in continuous cultures. *Microbiology*, 63(3):333–345, 1970. ISSN 1465-2080. doi: https://doi.org/10.1099/00221287-63-3-333. URL https://www.microbiologyresearch.org/content/journal/micro/10.1099/00221287-63-3-333.

[33] W. Scheifinger. Relationship of lactate dehydrogenase specificity and growth rate to lactate metabolism by *Selenomonas ruminantium*. *Applied Microbiology*, 30:916 – 921, 1975. URL https://api.semanticscholar.org/CorpusID:237234205.

[34] Robbert Kleerebezem and Mark C. M. van Loosdrecht. A generalized method for thermodynamic state analysis of environmental systems. *Critical Reviews in Environmental Science and Technology*, 40(1): 1–54, 2010. doi: 10.1080/10643380802000974. URL https://doi.org/10.1080/10643380802000974.

[35] Oliver Ebenhöh, Josha Ebeling, Ronja Meyer, Fabian Pohlkotte, and Tim Nies. Microbial pathway thermodynamics: Stoichiometric models unveil anabolic and catabolic processes. *Life*, 14(2):247, 2024. ISSN 2075-1729. doi: 10.3390/life14020247. URL https://www.mdpi.com/2075-1729/14/2/247.

[36] H. Streekstra, M. J. Teixeira de Mattos, O. M. Neijssel, and D. W. Tempest. Overflow metabolism during anaerobic growth of *Klebsiella aerogenes* NCTC 418 on glycerol and dihydroxyacetone in chemostat culture. *Archives of Microbiology*, 147(3):268–275, 1987. doi: 10.1007/BF00463487. URL https://doi.org/10.1007/BF00463487.

[37] C L Johnson, Y A Cha, and J R Stern. Citrate uptake in membrane vesicles of *Klebsiella aerogenes*. *Journal of Bacteriology*, 121(2):682–687, 1975. doi: 10.1128/jb.121.2.682-687.1975. URL https://journals.asm.org/doi/abs/10.1128/jb.121.2.682-687.1975.

[38] J. L. Snoep, M. Mrwebi, J. M. Schuurmans, J. M. Rohwer, and M. J. Teixeira de Mattos. Control of specific growth rate in *Saccharomyces cerevisiae*. *Microbiology*, 155(5): 1699–1707, 2009. ISSN 1465-2080. doi: https://doi.org/10.1099/mic.0.023119-0. URL https://www.microbiologyresearch.org/content/journal/micro/10.1099/mic.0.023119-0.

[39] Pranas Grigaitis, Douwe A. J. Grundel, Eunice van Pelt-KleinJan, Mirushe Isaku, Guixiang Xie, Sebastian Mendoza Farias, Bas Teusink, and Johan H. van Heerden. A computational toolbox to investigate the metabolic potential and resource allocation in fission yeast. *mSystems*, 7(4):e00423–22, 2022. doi: 10.1128/msystems.00423-22. URL https://journals.asm.org/doi/abs/10.1128/msystems.00423-22.

[40] J. Snay, J. W. Jeong, and M. M. Ataai. Effects of growth conditions on carbon utilization and organic by-product formation in *B. subtilis*. *Biotechnology Progress*, 5(2):63–69, 1989. doi: https://doi.org/10.1002/btpr.5420050207. URL https://aiche.onlinelibrary.wiley.com/doi/abs/10.1002/btpr.5420050207.

[41] Helena Albergaria, Ana Torrão, Timothy Hogg, and Francisco Gírio. Physiological behavior of *Hanseniaspora guilliermondii* in aerobic glucose-limited continuous culture. *FEMS yeast research*, 3:211–6, 05 2003. doi: 10.1016/S1567-1356(02)00187-3.

[42] Sergio Renilla, Vicente Bernal, Tobias Fuhrer, Sara Castaño-Cerezo, José Pastor, Jose Iborra, Uwe Sauer, and Manuel Cánovas. Acetate scavenging activity in *Escherichia coli*: Interplay of acetyl-CoA synthetase and the PEP-glyoxylate cycle in chemostat cultures. *Applied microbiology and biotechnology*, 93:2109–24, 09 2011. doi: 10.1007/s00253-011-3536-4.
